# Sox5 controls cell cycle progression in intermediate progenitor cells during dentate gyrus development

**DOI:** 10.3389/fcell.2026.1876208

**Published:** 2026-07-23

**Authors:** Paula Tirado-Melendro, Lingling Li, Cristina Medina-Menéndez, Pilar Jurado-Angulo, Pilar Rodríguez-Martín, Laura García-Redondo, Katarzyna Bilińska, Aixa V. Morales

**Affiliations:** 1 Instituto Cajal (CSIC), Madrid and Centro de Neurociencias Cajal (CSIC), Alcalá de Henares, Spain; 2 PhD Program in Neuroscience, Universidad Autónoma de Madrid-Cajal Institute, Madrid, Spain; 3 Department of Clinical Genetics, Faculty of Medicine, Collegium Medicum in Bydgoszcz, Nicolaus Copernicus University, Bydgoszcz, Poland

**Keywords:** adult neurogenesis, cell cycle, hippocampus, Lamb-Shaffer syndrome, neurogenesis, proliferation, Sox5, Sox6

## Abstract

The development of the dentate gyrus (DG) of the hippocampus is protracted over time in comparison with other brain regions such as hippocampal cornus ammoni or neocortex, extending over the first postnatal weeks. During DG postnatal development, neural stem cells (NSCs) will remain to generate the adult neurogenic niche that will sustain granule neuron (GN) production throughout life. NSCs in the DG divide to generate intermediate progenitor cells (IPCs), whose highly regulated dynamics of self-renewal or cell cycle exit decisions still remain poorly understood. Sox5 is a transcription factor (TF) essential for the establishment of adult NSCs, however, its potential role in Sox5 expressing IPCs during DG development remains unexplored. In this study, we demonstrate that conditional loss of Sox5 during embryonic development leads to critical alterations in cell proliferation and survival in IPCs. Specifically, following Sox5 loss, IPCs exhibit a shortening of S-phase duration in late postnatal and juvenile adult stages. Furthermore, these alterations in IPC cell cycle dynamics could be behind the defects in GN differentiation observed in Sox5-defective mice that ultimately leads to subtle morphological changes in DG architecture. Finally, we demonstrate that the additional loss of one Sox6 copy, a closely related TF to Sox5, lead to more profound disruptions in DG morphology than the one observed upon Sox5 loss. Overall, these findings point to a prominent role for Sox5 in combination with Sox6 in IPC cell cycle progression and GN maturation during postnatal DG development.

## Introduction

Unlike the majority of brain regions, the development of the DG of the mouse hippocampus continues into postnatal stages and involves the sequential migration of multipotent progenitors. DG progenitors originate around E11.5 from the dentate neuroepithelium, an area of the medial pallium, constituting the primary (1^ry^) matrix (Altman and Bayer, 1990). Then, supported by a scaffold composed of Cajal-Retzius cells and radial glial cells, these progenitors and their progeny migrate through the secondary (2^ry^) matrix towards the pial side of the cortex, towards the hippocampal fissure. In this area, they establish the tertiary (3^ry^) matrix, the only source of progenitors and mature neurons by early postnatal stages (Morales and Mira, 2019). The majority of GNs of the DG will be generated during the first two postnatal weeks, with a peak at birth, condensing in the two characteristic DG blades (Hatami et al., 2018; Snyder, 2019). Simultaneously, a subset of NSCs gradually enters quiescence to establish the adult NSC population (Berg et al., 2019; Noguchi et al., 2019; Medina-Menéndez et al., 2025) which is confined to the inner part of the granule cell layer (GCL), the subgranular zone (SGZ). Upon activation, these adult NSCs re-enter cell cycle to produce IPCs that after a few rounds of cell division generate new GNs throughout adulthood, in a process reminiscent of that of postnatal development (Altman and Das, 1965; Urban and Guillemot, 2014).

DG development is under the regulation of multiple factors that control different steps of the process and are also known modulators of general neurogenesis. WNT and BMP secreted proteins, for instance, are crucial early in development for DG patterning. Mice with mutations in critical components of these signalling pathways show a complete disruption of DG development and dramatic reductions in neurogenesis (Galceran et al., 2000; Lee et al., 2000; Zhou et al., 2004; Caronia et al., 2010; Choe et al., 2013). Deficiency in Gli3, a TF mediator of the Shh signalling pathway, whose targets include several members of the WNT cascade, causes a complete absence of the DG (Franz, 1994; Grove et al., 1998; Hasenpusch-Theil et al., 2012).

Several other TFs have been identified as key players in late DG development. Thus, NeuroD1 and Prox1 are essential for the survival, differentiation and maturation of GNs. Mutations in these genes result in the complete absence of the DG due to GN cell death (Miyata et al., 1999; Liu et al., 2000; Galeeva et al., 2007; Roybon et al., 2009; Lavado et al., 2010; Iwano et al., 2012). Murine models deficient in other TFs show milder phenotypes, indicating a more discrete function of these proteins. For instance, disruption of COUP-TFI function leads to DG dysmorphogenesis, specially along the ventral part of the axis, mainly due to loss of GNs and migratory defects (Flore et al., 2017; Parisot et al., 2017). Similar observations have been made regarding factors such as NFIX, (Heng et al., 2014), Tbr2 (Hodge et al., 2012, 2013), Ngn2 (Galichet et al., 2008), Bcl11b (Simon et al., 2012), ERK1/2 pathway signalling effectors (Vithayathil et al., 2015) and deubiquitylating enzyme USP9X (Oishi et al., 2016), in which although DG is apparent, its growth and morphology are impaired following their loss. Even though these studies analyse neurogenic progression, very few of them tackle proliferation and cell cycle dynamics of IPCs (Galichet et al., 2008; Hodge et al., 2012).

Some factors have been recently linked to NSC quiescence acquisition during early postnatal neurogenesis (Calatayud-Baselga et al., 2023; Pastor-Alonso et al., 2024; Medina-Menéndez et al., 2025), however, their role in other cell populations and general DG development has not been extensively explored. Sox5, a TF of the SoxD subfamily (Lefebvre, 2010), controls the initial NSC quiescence entry as well as the depth of the quiescent state during DG postnatal development (Medina-Menéndez et al., 2025). Moreover, in the adult DG, Sox5 and the related protein Sox6 are expressed in NSCs and IPCs and promote NSC activation and the generation of new neurons (Li et al., 2022). However, it is unclear if SoxD family members Sox5 and Sox6 could be involved in the control of cell cycle progression during DG development.

Here, we show that Sox5 and Sox6 display an overlapping expression pattern during DG development, being preferentially expressed in NSCs and IPCs, and are downregulated upon neuronal differentiation. Moreover, Sox5 conditional deletion during early embryonic brain development causes in young mouse an excess in IPC proliferation, a decrease in cell death and shortening of S-phase in IPCs. Futhermore, combined Sox5 full deficiency and Sox6 haploinsufficiency results in a more severe phenotype than that observed in Sox5-deficient mice. Overall, our results point to the relevance of both SoxD family members in the control of IPC cell cycle dynamics during DG development.

## Materials and methods

### Animals

All protocols involving animals were carried out in accordance with the guidelines of European Union (2010/63/UE) and Spanish legislation (53/2013, BOE no. 1337). Mice were housed with a standard control of a 12-h light/dark cycle and maintained in the animal facility at Cajal Institute, currently Centro de Neurociencias Cajal.

Sox5^fl/fl^ mice, in which coding exon 5 of Sox5 gene is flanked by *loxP* sites (Dy et al., 2008), and Sox6^fl/fl^ mice, generated by flanking the second coding exon with *loxP* sites (Lefebvre et al., 2007), were used to model Sox5 and Sox6 loss, respectively. In order to generate neural-specific conditional knockout mice, they were bred with Nestin-Cre mice (RRID:IMSR_JAX:003771) to generate control animals (Control): Nestin-Cre/Sox5/6^fl/+,^ Sox5/6^fl/fl^ or Sox5/6^fl/+^, Sox5 mutant mice: Nestin-Cre/Sox5^fl/fl^ (Sox5^null^), Sox6 mutant mice: Nestin-Cre/Sox6^fl/fl^ (Sox6^null^) and Sox5 and Sox6 mutant mice: Nestin-Cre/Sox5^fl/fl^/Sox6^fl/+^ (Sox5^null^ Sox6^het^). Both males and females of the strain C57B1/6 were used for genetic studies with no distinction.

### Thymidine analogues administration

Thymidine analogues were injected intraperitoneally to mice at a dose of 50 mg/kg for BrdU (10280879001, Roche), 42.5 mg/kg for CldU (C6891, Sigma-Aldrich) and 57.5 mg/kg for IdU (I7125, Sigma-Aldrich) to label S-phase cells. To label proliferating progenitors, brains were collected 1-h post-injection of a single administration of BrdU. For cell cycle assays, postnatal day 5 (P5), P14 and P30 mice received a single injection of BrdU 24 h prior tissue collection. To analyse cell cycle duration, P14 mice were injected with a single injection of CldU followed by an injection of IdU 3 h later. Tissue was collected 30 min after the last injection.

### Tissue preparation

Animals were transcardially perfused with cold 0.1 M phosphate buffer (PB) followed by 4% paraformaldehyde (PFA). Brains were postfixed with 4% PFA for 4 h at 4 °C, embedded in 30% agarose/sucrose (w/v) and coronally sectioned in 50 μm slices using a vibratome.

### Nissl staining

Vibratome sections were collected in 4–8 series, each one representing the whole anterior to posterior axis of the hippocampus with an interval distance of 200 μm or 400 μm. Sections representative of the whole hippocampus were mounted and air dried. Sections were then re-fixed first with cold 4% PFA for 5 min and washed with Milli-Q H_2_O for 1 min. Sections were stained with 0.5% Thionin in Milli-Q H_2_O for 2 min and immersed consecutively in 30%, 50%, 70%, 95% and 100% EtOH for 1 min to bleach the staining. After clearing sections in Xylene for 5 min, they were mounted with coverslips using DePeX. All samples were photographed using a ×10 Plan Nikon Objective in a stereomicroscope (Nikon).

### Immunofluorescence staining

For immunostaining, vibratome floating brain sections were permeabilized with 1% Triton X-100 in 0.1M PB for 1 h and blocked with 10% fetal bovine serum (FBS) and 0.25% Triton in 0.1M PB for 2 h at room temperature with rocking. Primary antibodies: BrdU (1:500, ab6326, Abcam), CldU (1:200, C6891, Sigma-Aldrich), DCX (1:500, ab18723, Abcam), GFAP (1:1000, Z0334, Dako), IBA1 (1:200, 019-19741, Wako), IdU (1:200, BD347580, BD Biosciences), Ki67 (1:500, 550609, Bd Biosciences), MCM2 (1:1000, 610701, Bd Biosciences), Nestin (1:250, NES8967981, Aves Labs), NeuN (1:500, MAB377, Merck Milipore), Prox1 (1:1000, MAB5654, Merck and 1:500, AF2727, R&D Systems), Sox2 (1:500, AF 2018, R&D Systems), Sox5 (1:500, A. Morales), Sox6 (1:1000, M. Wegner, and 1:10, ab30455, Abcam), Tbr2 (1:500, ab23345, Abcam) were prepared in incubation buffer (1% FBS, 0.25% Triton in 0.1M PB) and incubated overnight at 4 °C. Following three washes with washing buffer (0.1% Triton in 0.1M PB), immunoreactivity was detected using appropriate Alexa Fluor-conjugated secondary antibodies (1:1000) in incubation buffer for 2 h at room temperature. After three washes with washing buffer, sections were incubated with bisbenzimide (1:100, in 0.1M PB) for 2 min at room temperature and mounted using Fluoromount-G (Thermo-Fisher).

To detect thymidine analogue incorporation, DNA was denatured by incubating sections with 2N HCl for 15 min at 37 °C, followed by washes in 0.1M PB.

### Microscopy and image analysis

All immunofluorescence images were taken with direct SP5 and Stellaris confocal microscopes (Leica). Images of both left and right dorsal DG sections were captured with a z-step of 2 μm through at least 20 μm of each 50 μm sections by ×40 objective. At least 3 sections per mice of each genotype were analyzed for every analysis, selecting exclusively dorsal DG sections (−1.34 mm to −2.18 mm from bregma).

Counting was performed manually and blind using LAS X software (Leica). Quantitation was done counting the number of cells (at least 100 cells/marker) that expressed a cell-type specific maker in the general population of DG cells or among DG cells expressing a certain cell-type marker. Number of cells positive for a specific marker was normalized to the whole section (50 um) area (Galichet et al., 2008; Heng et al., 2014). For the 4 bin analysis of DCX^+^ cells, DG thickness was divided into 4 equally sized fractions and cells were counted in the suprapyramidal and infrapyramidal blade to avoid biases.

DG morphology analysis was done using Fiji ImageJ Software on images captured using a Thunder microscope (Leica). Measurements were performed on bisbenzimide stained sections of a minimum of 5 animals of each group. A minimum of 3 and a maximum of 8 sections only from dorsal DG (−1.34 mm to −2.18 mm from bregma) of each animal were used to measure DG area, blade length, GCL width and DG angle. For GCL width, three measurements were taken in the distal, medial, and proximal regions of the supra and infrapyramidal blades, and the average was calculated.

### Statistical analysis

The appropriate sample size (N) was determined based on similar published data (Li et al., 2022), using a minimum of 3 mice per condition. Statistical analyses and graphs were conducted in GraphPad Prism version 8 software using different tests with a significance level of at least p < 0.05. Thus, two-tailed unpaired Student’s t-test was used for most of statistical comparisons of two conditions and two-way ANOVA followed by Turkey’s multiple comparison tests, was used for comparison between more than two groups. Statistical details were included in each figure and figure legend. Data are presented as mean ± SEM. Significance is stated as follows: p < 0.05 (*), p < 0.01 (**), p < 0.001 (***), confidence intervals of 95%.

### Drawings

All the drawings in the Figures have been done using Inkscape 1.3.2.

## Results

### Sox5 loss alters DG morphology in young adult mice

**FIGURE 1:**
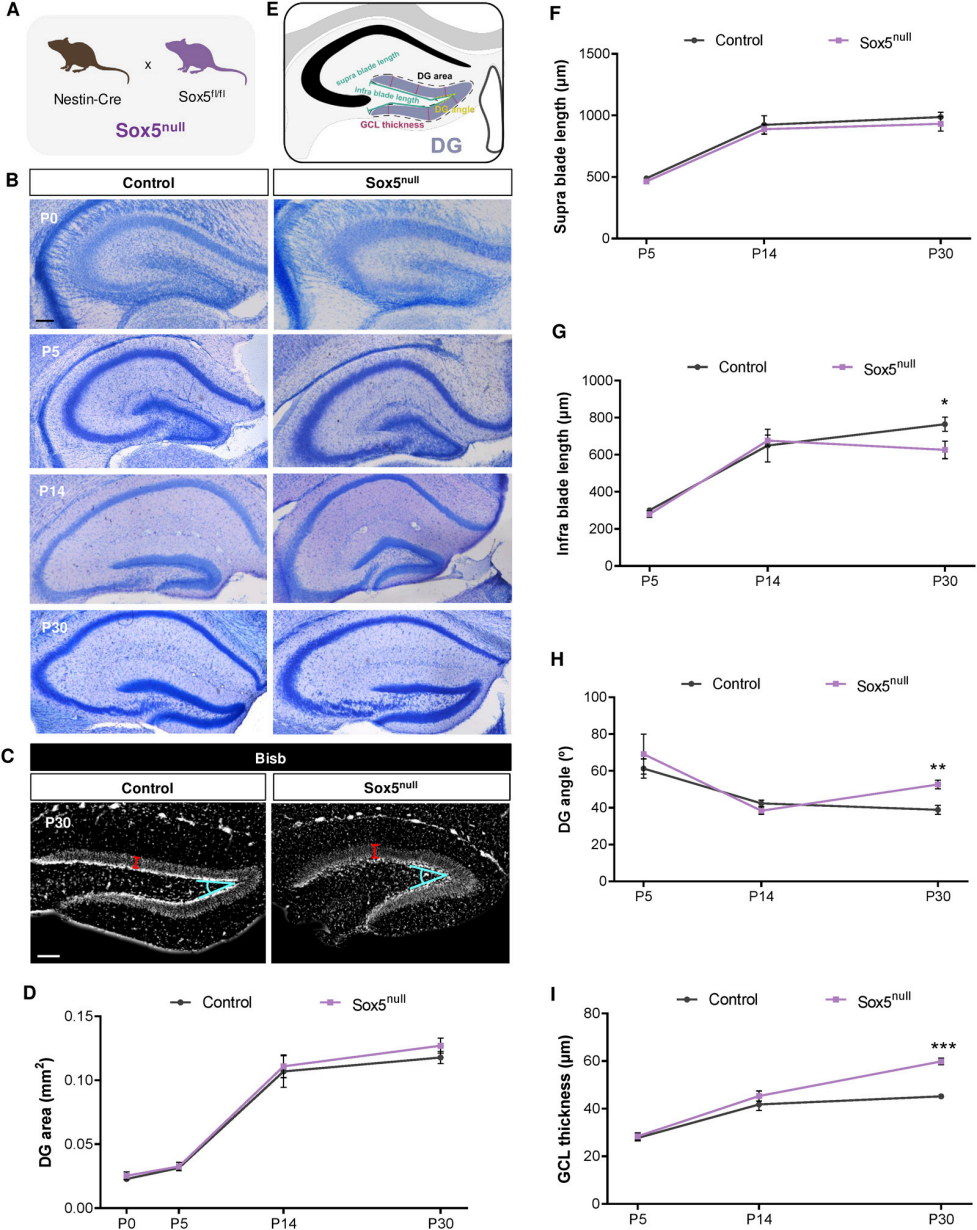
Sox5 loss alters DG morphology in young adult mice. **(A)** Generation of Sox5^null^ mice. **(B)** Nissl staining images showing DG morphology in brain coronal sections of Control and Sox5^null^ mice at the indicated stages. **(C)** Bisbenzimide staining confocal images showing DG morphology, GCL thickness (red), DG angle (blue) of Control and Sox5^null^ mice at P30. **(E)** Diagram showing the different DG parameters analyzed. Quantitation of DG area **(D)**, suprapyramidal blade length **(F)**, infrapyramidal blade length **(G)**, DG angle between blades **(H)** and GCL thickness **(I)** was performed in Control and Sox5^null^ mice at P0 (Control n = 6, Sox5^null^ n = 6), P5 (Control n = 3, Sox5^null^ n = 3), P14 (Control n = 3, Sox5^null^ n = 7) and P30 (Control n = 5, Sox5^null^ n = 6) exclusively in dorsal DG. In all graphs, data are mean value ± SEM. *p < 0.05; **p < 0.01 by unpaired Student’s t-test. Scale bars represent 100 µm in P0-P30 and 130 µm in P5 **(B,C)**.

During the development of the DG, the majority of NSCs express Sox5, a TF crucial for NSC quiescence entry (Medina-Menéndez et al., 2025). Moreover, around 10% of IPCs maintain high levels of Sox5 expression, both in the developing and the adult DG (Medina-Menéndez et al., 2025; Li et al., 2022). For that reason, we investigated if Sox5 conditional loss using Nestin-cre mediated recombination (Sox5^null^ mice; Medina-Menéndez et al., 2025) could have an impact in DG morphology and size during DG development (Figures 1A,B,E).

Nissl staining of coronal sections did not reveal gross morphological nor size alterations in Sox5^null^ DGs at developmental or adult stages (Figure 1B, Supplementary Figure S1). Further quantification of DG area using bisbenzimide staining did not show any differences between Control and Sox5^null^ mice from postnatal day 0 (P0) to P30 stages (Figures 1B–D). Accordingly, Control and Sox5-deficient mice exhibited similar suprapyramidal (Figures 1B,F) and infrapyramidal DG blade length (Figures 1B,G) at P5 and P14. However, at P30, we observed a decrease in Sox5^null^ infrapyramidal blade length (760.4 ± 32.94 µm in Control vs. 626.6 ± 47.41 µm in Sox5^null^; *P* = 0.022; Figures 1B,C,G). In addition, Sox5^null^ DG at P30 had a wider angle between supra and infrapyramidal blades compared to Controls (52.67 ± 2.33° vs. 38.91 ± 2.43°, respectively; *P* = 0.038; Figure 1B,C (blue),H) as well as a thicker GCL (45.35 ± 0.73 µm in Control vs. 59.86 ± 1.37 µm in Sox5^null^; *P* = 0.00002; Figures 1B,C (red),I).

In conclusion, these data indicate that developmental loss of *Sox5* induces subtle alterations in specific DG morphological traits in young adult mice, suggesting possible alterations in NSCs and/or IPC proliferation and in neurogenesis in Sox5^null^ mice.

### Sox5 loss impacts cell proliferation and survival in the DG during postnatal stages

Specific alterations in NSC proliferation upon Sox5 loss have been previously analysed in the context of adult neurogenic niches (Kurtsdotter et al., 2017; Li et al., 2022; Medina-Menéndez et al., 2025). In order to explore the possible alterations behind the changes in DG morphology observed at P30, we analysed the general proliferative and apoptotic state of DG progenitor neural cells during the first postnatal weeks in Sox5^null^ mice.

**FIGURE 2:**
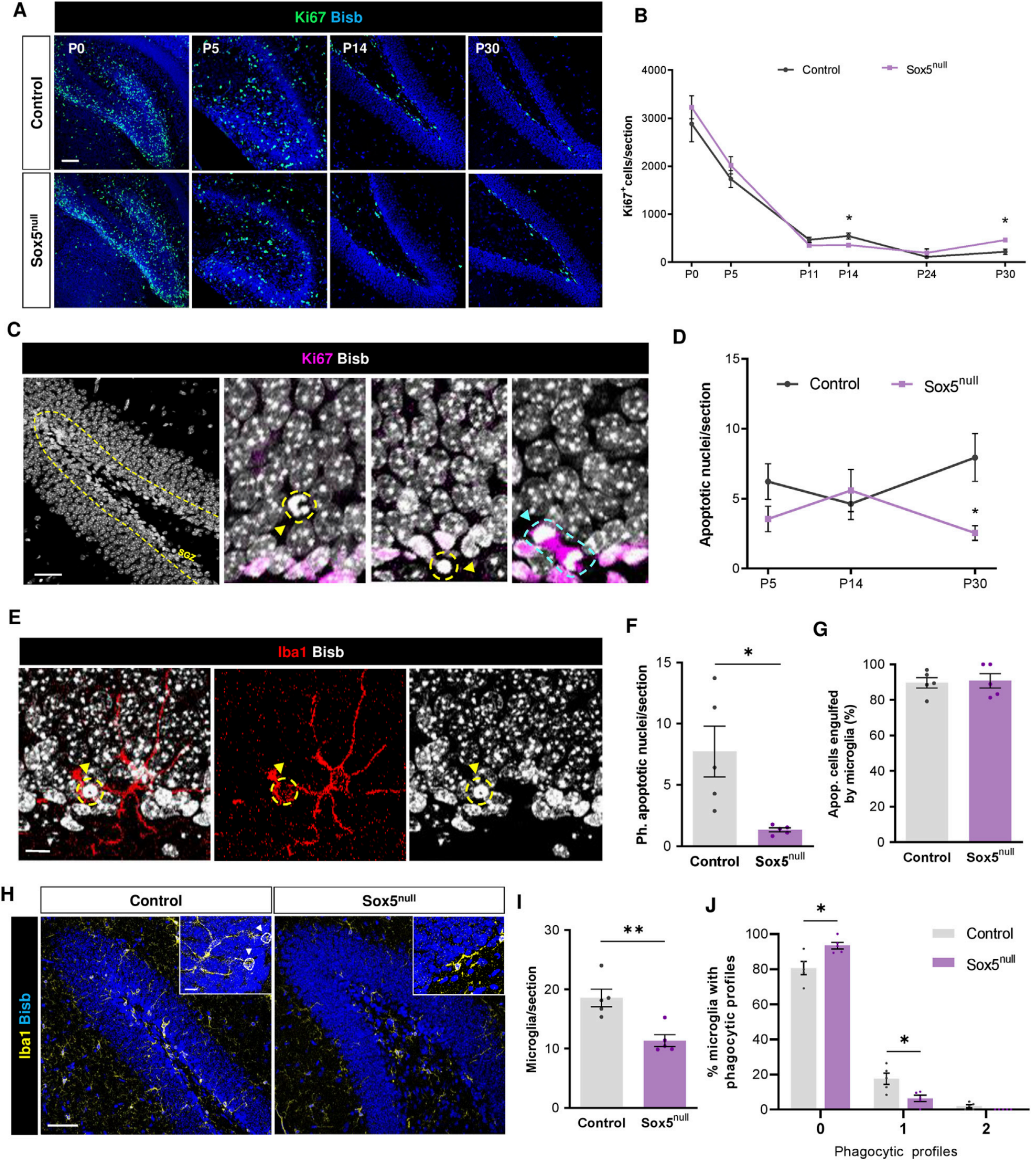
Sox5 loss impacts cell proliferation and survival in the DG during postnatal stages. **(A)** Confocal images showing Ki67^+^ proliferative cells in the DG of Control and Sox5^null^ mice at indicated stages. **(B)** Quantitation of Ki67^+^ proliferative cells in the DG per section at P0 (Control n = 3, Sox5^null^ n = 3), P5 (Control n = 3, Sox5^null^ n = 3), P11 (Control n = 4, Sox5^null^ n = 3), P14 (Control n = 5, Sox5^null^ n = 4), P24 (Control n = 5, Sox5^null^ n = 4) and P30 (Control n = 4, Sox5^null^ n = 3). **(C)** Confocal images showing apoptotic nuclei morphology in the SGZ (yellow arrow) vs. diving cell (blue arrow) of a P30 Control mouse. **(D)** Quantitation of apoptotic nuclei stained with bisbenzimide in the DG per section at P5 (Control n = 6, Sox5^null^ n = 5), P14 (Control n = 7, Sox5^null^ n = 4) and P30 (Control n = 4, Sox5^null^ n = 4). **(E)** Confocal images showing apoptotic nucleus (yellow arrow) being phagocytosed by microglia in the SGZ of P30 mice. Quantitation of phagocytosed apoptotic nuclei per section **(F)** and of the % of apoptotic cells engulfed by microglia (engulfed nuclei % over total apoptotic nuclei) **(G)** in P30 Control and Sox5^null^ mice. **(H)** Confocal images showing the resident microglia in P30 Control and Sox5^null^ mice. Magnification of Iba1^+^ cells with two phagocytic profiles (Control) and no phagocytic profiles (Sox5^null^). Quantitation of the number of microglia per section **(I)** and their phagocytic capacity (% of microglia with 0–2 phagocytic profiles) **(J)** in P30 Control and Sox5^null^ mice. At least 3 animals were analyzed for each group. In all graphs, data are mean value ± SEM. *p < 0.05; **p < 0.01 by unpaired Student’s t-test. Scale bars represent 50 µm in P5-P30 **(A)** and in DG **(H)**, 40 µm in DG **(C)**, 100 µm in P0 **(A)** and 10 µm in images with higher magnification **(C,E,H)**.

Cell division decreases abruptly in the DG during the first postnatal weeks, when NSCs enter quiescence for the first time (Berg et al., 2019; Noguchi et al., 2019; Medina-Menéndez et al., 2025) and the majority of adult GNs are generated (Bayer and Altman, 1974; Bond et al., 2021). We used Ki67 labelling, a cell cycle marker expressed from the end of G1 phase until the end of mitosis in NSCs and IPCs (Miller et al., 2018). We observed that the proliferative cell fraction was very similar in Control and in Sox5^null^ mice at P5 (1735.42 ± 177.60 and 2016.67 ± 181.48 cells/section, respectively; Figures 2A,B) and at P11 (465.79 ± 56.48 in Control vs. 350.46 ± 38.94 cells/section in Sox5^null^, Figure 2B). However, by P14, Sox5^null^ mice showed a significant 35% reduction in the number of Ki67^+^ cells with respect to Control mice (545.83 ± 64.57 in Control vs. 357.29 ± 13.95 cells/section in Sox5^null^; *P* = 0.0419; Figures 2A,B). By contrast, at P30, when all the proliferating neural cells are located in the SGZ (Figure 2A) and DG development is completed, the total number of Ki67^+^ cells in the SGZ is significantly increased in Sox5^null^ mice compared to Control mice (461.92 ± 22.72 vs. 217.16 ± 56.19 cells/section, respectively; *P* = 0.0166; Figures 2A,B). At an intermediate state, at P24, a similar number of Ki67^+^ cells were observed between Control and Sox5-deficient mice (110.28 ± 30.68 in Control vs. 192.69 ± 83.61 cells/section in Sox5^null^; Figure 2B), suggesting a transitional state in IPC proliferation from the decrease at P14 and the increase observed at P30. In conclusion, there is a temporal increase in proliferation in DG cells at P30 in Sox5^null^ mice, which could be in part responsible for the increase in GCL thickness observed.

Apoptotic cell death is another major process that could directly affect the final number of generated GNs. Apoptotic cells show a distinct nuclear morphology, with DNA condensation or nuclear fragmentation (bisbenzimide staining; Figure 2C) that is coincident with activated caspase 3 labelling (Sierra et al., 2010). We observed that the number of apoptotic cells was comparable in Control and Sox5^null^ mice at P5 (6.22 ± 1.28 vs. 3.55 ± 0.9 cells/section, respectively; Figure 2D) and P14 (4.55 ± 1.60 vs. 5.58 ± 1.52 cells/section, respectively; Figure 2D). However, Sox5^null^ mice showed a decrease in the fraction of apoptotic cells with respect to Control mice by P30 (7.94 ± 1.71 in Control vs. 2.54 ± 0.63 cells/section in Sox5^null^; *P* = 0.0236; Figure 2D). As apoptotic cells are rapidly engulfed by surrounding microglia (Sierra et al., 2010; Figure 2E), we performed microglial staining using Iba1 marker in P30 mice. In line with the previous results, Sox5^null^ showed fewer apoptotic nuclei undergoing phagocytosis compared with Control animals (1.334 ± 0.17 and 7.73 ± 2.07 cells/section, respectively; *P =* 0.0364; Figure 2F). However, this decrease in phagocytosis was not associated with microglia clearance efficiency, as the percentage of apoptotic cells being engulfed was similar between Control and Sox5^null^ mice (89.61% ± 3.03% and 90.69% ± 3.99%, respectively; Figure 2G). Consistent with the previous cell death results, we observed a decrease in microglia cell number in Sox5^null^ mice (18.57 ± 3.30 cells/section in Control vs. 11.37 ± 1.01 cells/section in Sox5^null^; *P* = 0.038; Figures 2H,I). Furthermore, we analysed microglia’s efficiency by calculating their phagocytic capacity as the proportion of microglia with one or more phagocytic processes, each containing one apoptotic cell (Sierra et al., 2010; Beccari et al., 2018). We observed that Sox5-deficient mice showed a decrease in phagocytic capacity compared to Control animals. Specifically, Sox5^null^ mice exhibited a higher percentage of microglia with no phagocytic profiles (80.55% ± 3.70% in Control vs. 93.35% ± 1.94% in Sox5^null^; *P* = 0.015; Figures 2H,J) and a decrease in the percentage of processes phagocytosing one (17.50% ± 3.20% in Control vs. 6.40% ± 1.82% in Sox5^null^; *P* = 0.017; Figure 2J) or two apoptotic cells (1.95% ± 0.88% in Control vs. 0% ± 0% in Sox5^null^; *P* = 0.057; Figures 2H,J), which reflects the lower presence of microglia cells with one or more phagocytic processes engulfing an apoptotic cell. However, we could not define the identity of the dying cells by immunohistochemistry as it has been widely demonstrated that there is a general protein or antigenicity loss in apoptotic cells (Odin et al., 2001; Unal-Çevik et al., 2004).

These data demonstrate that the difference in DG size in Sox5^null^ mice by P30 is probably due to a combination of an increase in cell proliferation and a reduction in cell death, whereas there are not obvious alterations in microglia capacity to eliminate DG apoptotic cells in Sox5^null^ mice.

### Sox5 is required to control IPC number during DG development

In the developing DG, active NSCs and IPCs are the main cell populations engaged in cell cycle progression, the latter generated through neurogenic divisions from NSCs. We had previously shown that the NSC pool remains unchanged in Sox5^null^ mice during the first four postnatal weeks (Medina-Menéndez et al., 2025), therefore we evaluated IPC number, the other proliferative cell population that could be responsible for the proliferation differences found in Sox5*-*deficient mice.

**FIGURE 3:**
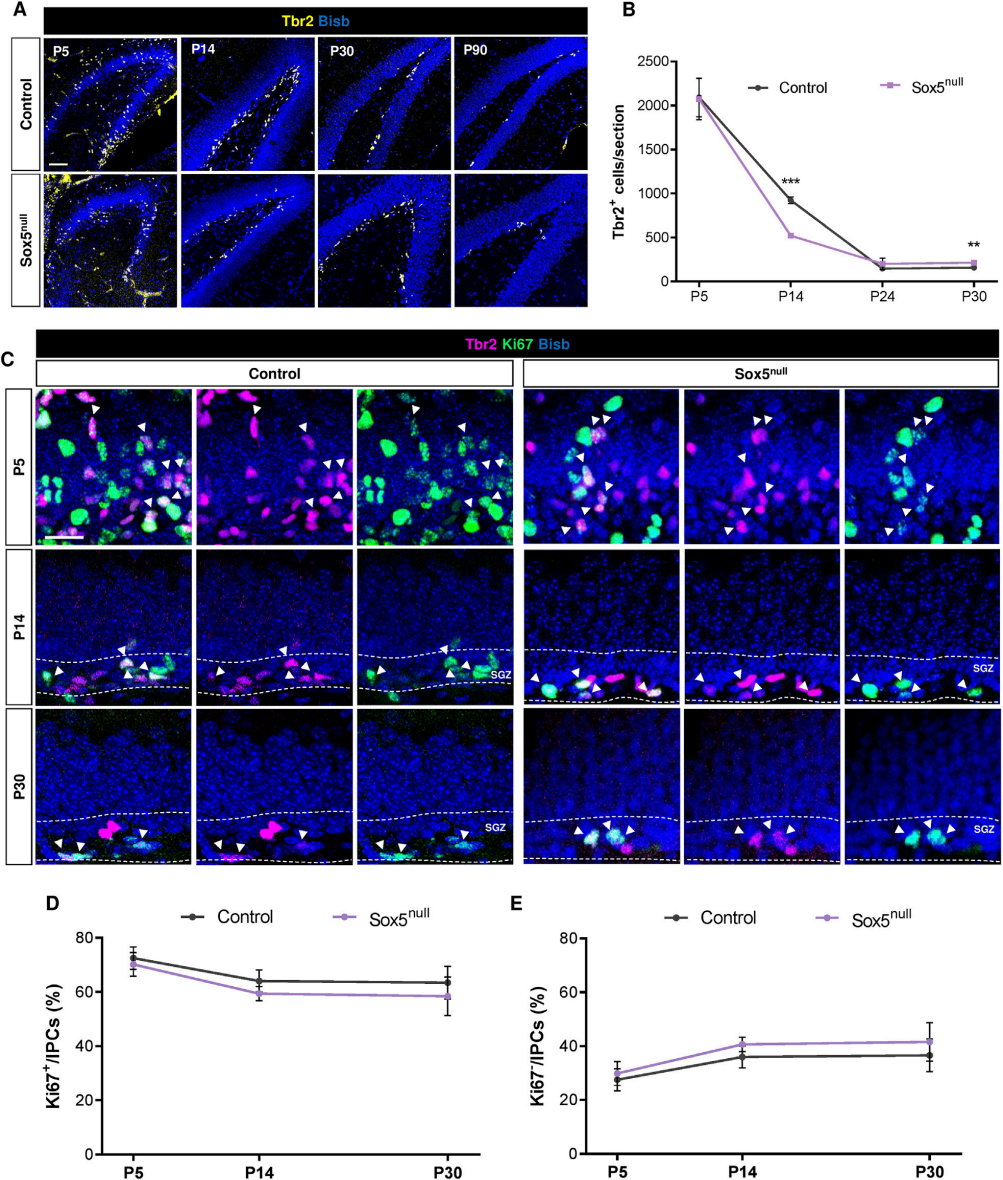
Sox5 is required to control IPC number during DG development. **(A)** Confocal images showing Tbr2^+^ IPCs in the DG of Control and Sox5^null^ mice at indicated stages. **(B)** Quantitation of Tbr2^+^ IPCs in the DG per section at P5 (Control n = 3, Sox5^null^ n = 3), P14 (Control n = 3, Sox5^null^ n = 3), P24 (Control n = 3, Sox5^null^ n = 3) and P30 (Control n = 4, Sox5^null^ n = 3). **(C)** Confocal images showing Tbr2 and Ki67 immunostaining in the DG of Control and Sox5^null^ mice at indicated stages. White arrows indicate proliferative Tbr2^+^ IPCs. Quantitation of proliferative Ki67^+^ IPCs (KI67^+^ Tbr2^+^ % over total Tbr2^+^ cells) **(D)** and non-proliferative Ki67^-^ IPCs (KI67^-^ Tbr2^+^ % over total Tbr2^+^ cells) **(E)** in the DG of Control and Sox5^null^ mice at P5 (Control n = 3, Sox5^null^ n = 3), P14 (Control n = 5, Sox5^null^ n = 5) and P30 (Control n = 3, Sox5^null^ n = 4). In all graphs, data are mean value ± SEM. *p < 0.05; **p < 0.01, ***p < 0.001 by unpaired Student’s t-test. Scale bars represent 50 µm **(A)** and 20 µm **(C)**.

In a similar way to what we had described for the dynamics of the growing fraction of neural progenitor cells along time (Figure 2B), the population of neuronal-fate committed IPCs (Tbr2^+^ cells) is sharply reduced from P5 to P14 (Figures 3A,B) in Control mice, as they progress through differentiation. During this period Tbr2^+^ cells, initially distributed throughout the whole DG, hilus and ML, get restricted to the SGZ area. This reduction in the number of IPCs continued in the transition from developmental to adult SGZ, from P14 to P30 (Figures 3A,B).

At P5, Sox5^null^ mice showed a similar number of Tbr2^+^ IPCs in DG compared with Control mice (2074.07 ± 234.84 vs. 2093.52 ± 219.84 cells/section, respectively; Figures 3A,B). However, by P14, we detected an abrupt 44% reduction in the number of IPCs in Sox5^null^ mice with respect to Control mice (520.76 ± 25.04 vs. 923.48 ± 36.96 cells/section, respectively; *P* = 0.0008; Figures 3A,B), which matched the general reduction in proliferation observed at P14 (Figure 2B). By contrast, consistently with the increase of Ki67^+^ cells in P30 Sox5^null^ animals (Figure 2B), we observed a 36% increase in the number of total IPCs in Sox5^null^ mice at P30 with respect to Control mice (213.83 ± 3.82 vs. 156.89 ± 10.01 cells/section, respectively; *P* = 0.0056; Figures 3A,B). At an intermediate state at P24, Sox5^null^ mice showed a similar number of Tbr2^+^ cells compared with Control mice (146.18 ± 13.23 in Control vs. 200.82 ± 63.56 in Sox5^null^; Figure 3B).

As the number of IPCs could be conditioned by their proliferation rate, we analysed the number of Ki67^+^ Tbr2^+^ cells among the IPC Tbr2^+^ population and observed that the proliferating fraction in Sox5^null^ mice was comparable to the one observed in Control mice at P5 (70.177% ± 4.43% vs. 72.49% ± 4.14%, respectively), P14 (59.39% ± 2.67% vs. 64.03% ± 4.09%, respectively) and P30 (58.44% ± 7.12% vs. 63.41% ± 6.07%, respectively; Figures 3C,D). Correspondingly, the complementary population of Ki67^-^ Tbr2^+^ remained similar between Control and Sox5^null^ mice at these stages (Figures 3C,E). These data indicate that there are no differences in the proliferative fraction of IPCs between Control and Sox5^null^ mice.

In summary, in the DG of Sox5^null^ mice there are profound and dynamic changes during postnatal development, with a temporal reduction in the number of IPCs by P14 and a sustained increase in IPCs from young to mature Sox5^null^ adult mice. Nevertheless, these alterations in Sox5-deficient mice are not caused by changes in the proliferative fraction of IPCs, that remains stable.

### Sox5 loss results in S-phase shortening in IPCs during late DG postnatal development

**FIGURE 4:**
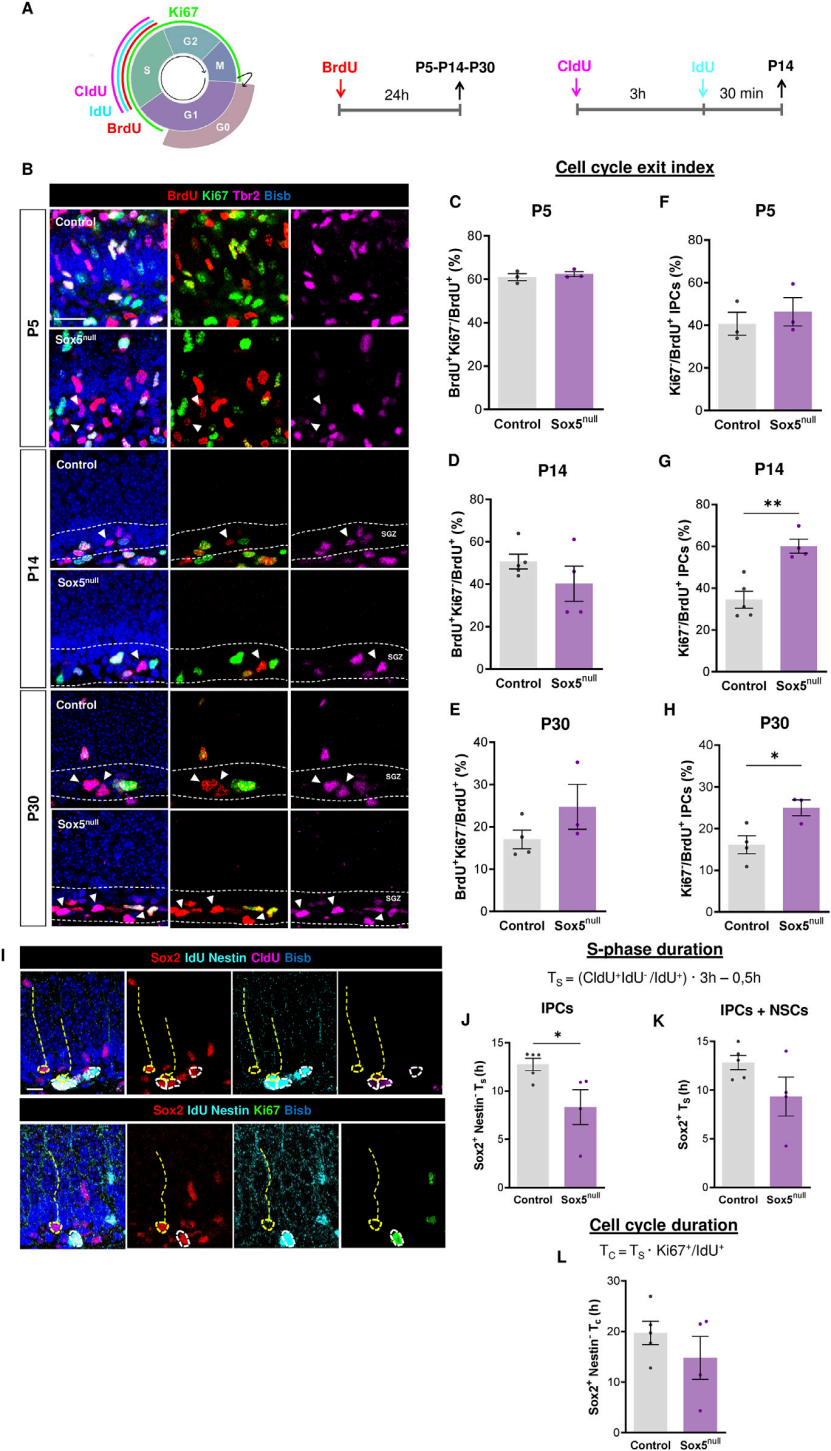
Sox5 loss results in S-phase shortening in IPCs during late DG postnatal development. **(A)** Scheme of thymidine analogue pulse labelling experiments for the analysis of cell cycle dynamics. For cell cycle exit index, one injection of BrdU was administrated 24 h before perfusion. For the estimation of cell cycle duration, one injection of CldU followed by a IdU injection 3 h later **(B)** Confocal images showing BrdU, Ki67 and Tbr2 staining in the DG of Control and Sox5^null^ mice at indicated stages. White arrows indicate Ki67^-^ BrdU^+^ Tbr2^+^ IPCs. **(C,D,E)** Quantitation of the % of Ki67^-^ cells in BrdU^+^ cells in P5, P14 and P30 mice, respectively. **(F,G,H)** Quantitation of the % of Ki67^-^ cells in BrdU^+^ Tbr2^+^ IPCs in P5, P14 and P30 mice, respectively. **(I)** Confocal images showing IdU, CldU, Nestin and Sox2 staining in the DG of Control and Sox5^null^ P14 mice. Quantitation of the S phase in Sox2^+^Nestin^−^ cells **(J)**, Sox2^+^ cells **(K)** and total cell cycle **(L)** duration of Control and Sox5^null^ P14 mice. At least 3 animals were analysed for each group. In all graphs, data are mean value ± SEM. *p < 0.05; **p < 0.01; by unpaired Student’s t-test. Scale bars represent 20 µm **(B)** and 10 µm **(I)**.

In order to explore if IPC number differences found in Sox5^null^ mice could be caused by an alteration in IPC cell cycle progression, we performed 24-h BrdU pulse-chase experiments in P5, P14 and P30 mice (Figure 4A), a time window that minimizes additional IPC cell divisions that could contribute to BrdU dilution, followed by Ki67 staining (Kee et al., 2002; Miller et al., 2018). In Control mice the cell cycle exit index (BrdU^+^Ki67^−^/BrdU^+^) decreased over time, from 61% ± 1.61% at P5 (Figures 4B,C), to a 50% ± 3.46% at P14 (Figures 4B,D) and abruptly diminished to 17% ± 2.20% at P30 (Figures 4B,E). A similar decrease was observed when analyzing the IPC population (BrdU^+^ Ki67^-^/BrdU^+^ IPCs) from 40.72% ± 5.36% at P5 (Figures 4B,F), 34.51% ± 4.05% at P14 (Figures 4B,G) to a 16.11% ± 2.16% at P30 (Figures 4B,H). This reduction could reflect either a decrease in cell cycle exit and differentiation in later stages of development or an indication of a progressive lengthening of the cell cycle during the first postnatal month. Consistently, it has been widely reported that cell cycle length increases in neural progenitors with time during central nervous system development, which has been linked to differentiation (Costa et al., 2011; Hardwick et al., 2015).

Cell cycle exit index showed similar values in the pool of proliferating cells in the DG of Sox5^null^ mice and Control mice at P5 (62.41% ± 1.51% and 61.00% ± 1.61%, respectively; Figures 4B,C), P14 (40.22% ± 8.27% and 50.70% ± 3.46%, respectively; Figures 4B,D) and P30 (24.72% ± 5.32% and 17.06% ± 2.20%, respectively; Figures 4B,E). When analyzing exclusively the IPC Tbr2^+^ population, we found no differences at P5 in Sox5^null^ mice (40.72% ± 5.36% in Control vs. 46.31% ± 6.64% in Sox5^null^; Figures 4B,F). However, P14 Sox5-deficient mice showed an increase in cell cycle exit index with respect to Control mice (BrdU^+^ Ki67^-^/BrdU^+^ IPCs, 34.51% ± 4.05% in Control vs. 60.10% ± 3.35% in Sox5^null^; *P* = 0.0022; Figures 4B,G) that was maintained at P30 (16.11% ± 2.63% in Control vs. 24.98% ± 1.95% in Sox5^null^; *P* = 0.0324; Figures 4B,H), suggesting alterations in cell cycle progression in Sox5^null^ IPCs.

As Tbr2^+^ IPCs are mostly mitotic neural progenitors, the differences observed in the ratio of Ki67^-^/BrdU^+^ IPCs between Control and Sox5^null^ mice at P14 and P30 could be reflective either of an increase in cell cycle exit or alterations in cell cycle length. To clarify this question, we resorted to the double thymidine analogue method (Nowakowski et al., 2002; Llorens-Martin et al., 2010; Ponti et al., 2013) and performed an initial injection of CldU in P14 mice, followed by a second injection of IdU 3 h later. After 30 min, mice were culled (Figure 4A). Consequently, CldU^+^ cells that were not labeled by IdU at the time of sacrifice would have left S phase in the 3-h interval between injections. With this approach, S-phase duration (T_S_) can be estimated, as the ratio of CldU^+^IdU^−^/IdU^+^ equals 3 h/(T_S_ + 0.5 h). Additionally, we could calculate total cell cycle duration (T_C_) from the ratio of cells in S phase to the entire proliferative population (IdU^+^/Ki67^+^), which is proportional to T_S_/T_C_ (Ponti et al., 2013).

To evaluate the duration of the S-phase specifically on IPCs, we analysed the Sox2^+^ Nestin^−^ cell population (Figure 4I, white). Sox2 is present mostly in NSCs and some IPCs, while Nestin is exclusively found on NSCs (Figure 4I, yellow). Therefore, the analysis of Sox2^+^ Nestin^−^ cells located in the SGZ would preferentially include IPCs, even though we cannot discard sparse NSC contribution. The estimation of T_S_ revealed a shortening of S-phase in P14 Sox5^null^ mice (12.76 ± 0.65 h in Control vs. 8.33 ± 1.82 h in Sox5^null^; *P* = 0.0396; Figures 4I,J) compared to Controls, whose T_S_ was similar to what had been previously described (Brandt et al., 2012). The analysis of Sox2^+^ cells, which would include both NSCs and IPCs showed no differences between Control and Sox5^null^ mice (12.32 ± 0.74 h in Control vs. 8.83 ± 1.99 h in Sox5^null^; Figures 4I,K), highlighting that the alteration in cell cycle length is exclusive to the IPC population. However, although we found a tendency towards a shortening in T_C_ in IPCs in Sox5-deficient mice with respect to Control mice, the differences were not statistically significant (14.82 ± 4.25 h and 19.72 ± 2.31 h, respectively; Figures 4I,L).

Collectively, these results point to a role for Sox5 in IPC cell cycle progression, ensuring the appropriate length of S-phase.

### New neuron generation and distribution are impaired following Sox5 loss

**FIGURE 5:**
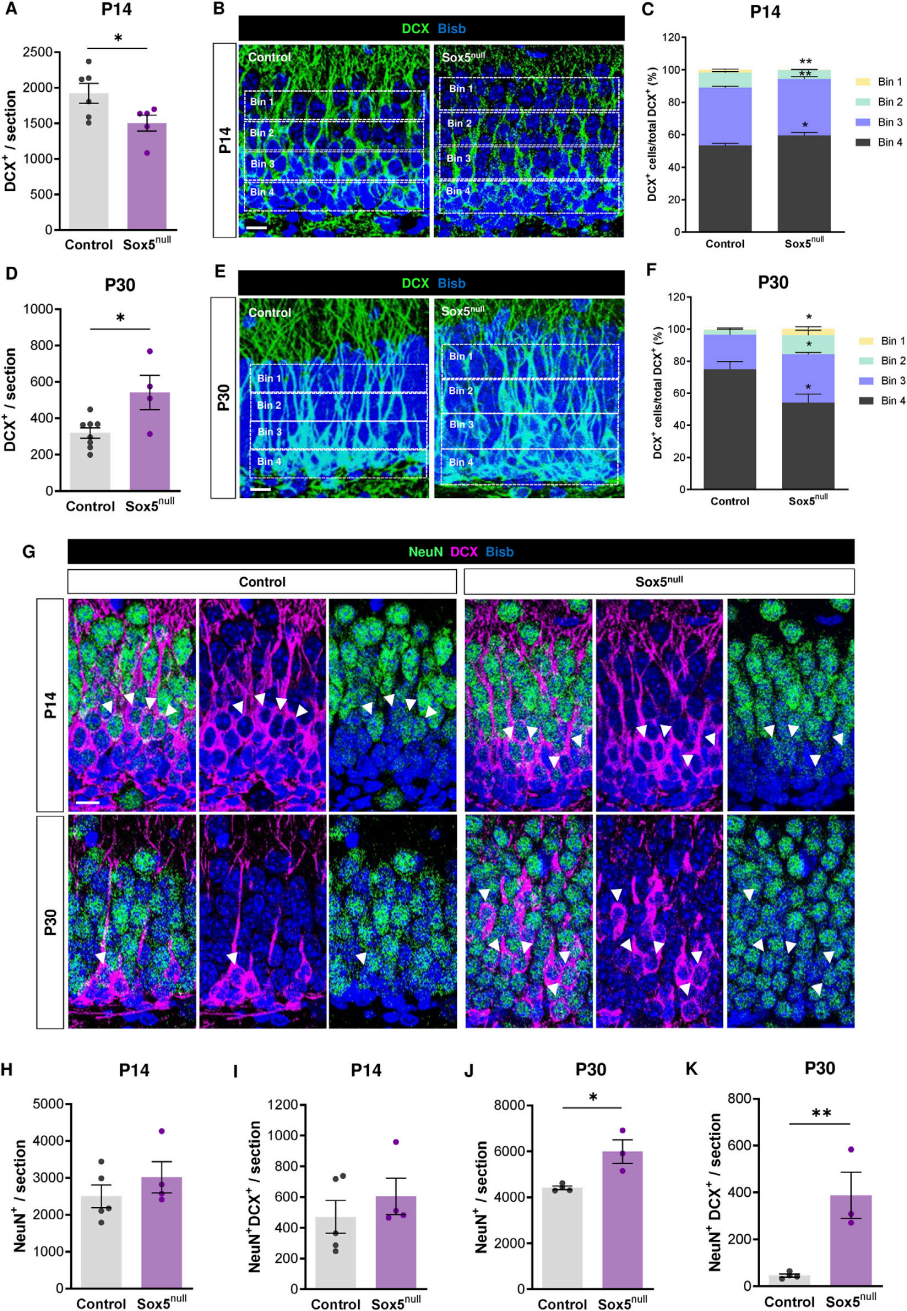
Sox5 loss alters new neuron production and location. Quantitation of DCX^+^ cells per section in the DG of Control and Sox5^null^ P14 **(A)** and P30 **(D)** mice. Confocal images showing DCX staining in the DG of Control and Sox5^null^ P14 **(B)** and P30 **(E)** mice with the bin scheme analysis. Quantitation of the % DCX^+^ cells in each bin (DCX^+^ in bin % over total DCX^+^ cells) in the DG of Control and Sox5^null^ P14 (Control n = 6, Sox5^null^ n = 5) **(C)** and P30 (Control n = 8, Sox5^null^ n = 4) **(F)** mice. **(G)** Confocal images showing NeuN and DCX staining in the DG of P14 and P30 Control and Sox5^null^ mice. White arrows indicate DCX^+^NeuN^+^ cells. Quantitation of NeuN^+^ cells per section in the DG of Control and Sox5^null^ P14 **(H)** and P30 **(J)** mice. Quantitation of the % of NeuN^+^ cells in DCX^+^ cells in P14 **(I)** and P30 **(K)** mice. At least 3 animals were analyzed for each group. In all graphs, data are mean value ± SEM. *p < 0.05; **p < 0.01; by unpaired Student’s t-test. Scale bars represent 10 µm.

Following cell cycle exit, IPCs differentiate into immature GNs that undergo maturation and integrate into hippocampal circuits. In order to evaluate the potential alterations in differentiation followed by IPC T_S_ shortening in Sox5^null^ mice, we analysed the immature neuron DCX^+^ population. Similarly to what we had previously described (Medina-Menéndez et al., 2025), we observed a decrease in the number of DCX^+^ cells in P14 Sox5^null^ mice (1923 ± 139.0 in Control vs. 1505 ± 112.7 cells/section in Sox5^null^; *P* = 0.049; Figure 5A). In contrast, P30 Sox5^null^ mice had a higher number of DCX^+^ cells compared to Control animals, reflective of an increase in neurogenesis (541.2 ± 93.83 vs. 318.3 ± 29.00 cells/section, respectively; *P* = 0.0146; Figure 5D).

To further assess possible defects in differentiation, we addressed immature neuron distribution through the DG layers. In late postnatal and adult mice, newly generated immature neurons would remain close to the SGZ first and then migrate towards the molecular cell layer as maturation progresses. We divided the DG in four equally sized bins and quantified DCX^+^ cell number in each bin (Figures 5B,E). DCX^+^ cells of P14 Sox5^null^ mice were predominantly located closer to the SGZ, with a 59.53% ± 1.69% of DCX^+^ cells in bin 4 compared to 53.28% ± 1.31% in Control animals (*P* = 0.025; Figures 5B,C). Accordingly, mutant mice had less DCX^+^ cells in bin 2 (9.19% ± 0.77% in Control vs. 5.47% ± 0.45% in Sox5^null^; *P* = 0.006) and bin 1 (1.93% ± 0.39% in Control vs. 0.27% ± 0.16% in Sox5^null^; *P* = 0.009; Figures 5B,C) than Controls. By P30, Sox5^null^ mice had a broader distribution, with less immature neurons in bin 4 (74.95% ± 4.51% in Control vs. 53.97% ± 4.79% in Sox5^null^; *P* = 0.024; Figures 5E,F) and aberrant displacement to bin 2 (2.92% ± 1.16% in Control vs. 11.74% ± 2.77% in Sox5^null^; *P* = 0.010; Figures 5E,F) and bin 1 (0.26% ± 0.18% in Control vs. 3.95% ± 1.22% in Sox5^null^; *P* = 0.004; Figures 5E,F). Thus, loss of Sox5 expression provokes changes in neurogenesis that follow the alterations in IPC numbers (Figures 3A,B) and in the migration or maturation of the immature DCX^+^ neurons.

In order to evaluate the progression of the maturation process, we then analysed NeuN expression, a marker of mature GNs. We found similar numbers of NeuN^+^ cells in P14 Sox5^null^ mice compared to Control animals (3216.90 ± 528.70 vs. 2867.07 ± 370.60 cells/section, respectively; Figures 5G,H) and of neurons in an intermediate maturation state reflected by DCX^+^ NeuN^+^ expression (Figures 5G,I). Interestingly, P30 Sox5 mutant mice showed an increase in NeuN^+^ neurons compared with Controls (5989.42 ± 511.06 vs. 4414.40 ± 76.86 cells/section, respectively; *P* = 0.016; Figures 5G,J) and a striking increase in the DCX^+^ NeuN^+^ intermediate population, suggesting an additional defect in the maturation process (8.81% ± 2.62% in Control vs. 28.81% ± 1.32% in Sox5^null^; *P* = 0.09; Figures 5G,K).

To sum up, Sox5^null^ mice show aberrant neurogenesis and DCX^+^ cell distribution across the DG layers at P14 and P30. Moreover, those alterations in migration and the striking increase in double DCX^+^ NeuN^+^ neurons at P30 could be a reflect of the shortened T_S_ of IPCs at P14, crucial timing for establishing the correct differentiation and maturation program. Moreover, those alterations in the program of neuronal maturation and migration could have an important impact on the circuit integration of the newborn neurons in Sox5^null^ mice.

### Sox6 loss does not have an impact on the proliferation of DG neural progenitors

**FIGURE 6:**
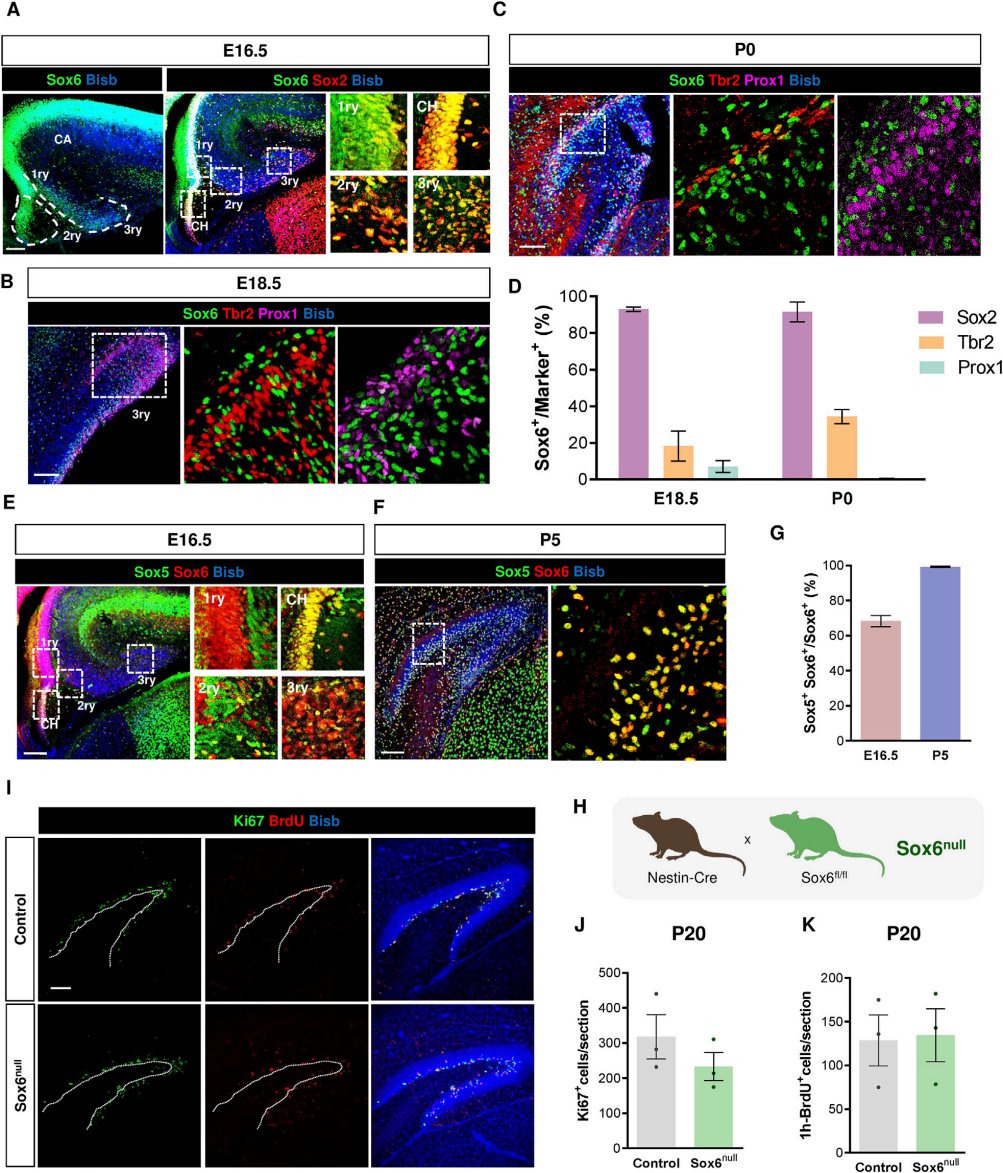
Sox6 loss does not have an impact on the proliferation of DG neural progenitors. **(A–C)** Confocal images showing Sox6 coexpression with Sox2, Tbr2 and Prox1 at indicated stages. **(D)** Quantitation of the % of Sox6^+^ cells among Sox2^+^, Tbr2^+^ and Prox1^+^ cell population in the 3ry matrix of the DG of E18.5 (n = 3) and P0 (n = 3) Control mice. **(E,F)** Confocal images showing Sox5 coexpression with Sox6 at indicated stages. **(G)** Quantitation of the % of Sox5^+^ cells among the Sox6^+^ cell population in the DG of E16.5 (n = 3) and P5 (n = 3) Control mice. **(H)** Generation of Sox6^null^ mice. **(I)** Confocal images showing Ki67^+^ and 1-h pulse BrdU^+^ cells in the DG of Control and Sox5^null^ P20 mice. **(J)** Quantitation of Ki67^+^ proliferative cells per section in the DG of Control and Sox5^null^ P20 mice. **(K)** Quantitation of BrdU^+^ proliferative cells per section following a 1-h pulse of BrdU in the DG of Control and Sox5^null^ P20 mice. At least 3 animals were analyzed for each group. Scale bars represent 100 μm and 30 µm **(B,E)**, 20 µm **(C,F)** and 15 µm **(A)** in images with higher magnification.

Sox5 and Sox6, are TFs that share overlapping functions when expressed in the same cell type, such as in oligodendrocytes precursors (Stolt et al., 2006) or in NSCs in the adult hippocampus (Li et al., 2022). During DG development, we observed Sox6 expressing cells in all three matrixes as well as in the cortical hem during embryonic (E16.5 and E18.5) and postnatal DG development (P0; Figures 6A–C). A detailed analysis revealed that 93% ± 1.15% and 91.55% ± 3.85% of Sox2^+^ cells coexpressed Sox6 in the 3ry matrix at E18.5 and in the future site of the DG at P0, respectively. However, we estimated that only 18.27% ± 8.24% of Tbr2^+^ IPCs and 7.05% ± 3.24% of Prox1^+^ GNs were also positive for Sox6 at E18.5 (Figures 6B,D). Similarly, we found a low percentage of Sox6^+^ Tbr2^+^ cells (34.38% ± 3.84%) and very scarce Sox6^+^ Prox1^+^ cells (0.33% ± 0.33%) in P0 Control mice (Figures 6C,D). These results strongly suggest that Sox6 is expressed in NSCs and IPCs and its expression is lost with neural differentiation. Given the similarities with Sox5 expression pattern along the neurogenic cascade (Medina-Menéndez et al., 2025), we then examined Sox6 and Sox5 coexpression in embryonic and early postnatal stages. Both in E16.5 and P5 mice, we found a high percentage of Sox6^+^ cells that also expressed Sox5 (68.3% ± 3.14% and 99.23% ± 0.39%, respectively; Figures 6E–G), evidencing their coexpression in most of the cells of the developing DG, except for embryonic 1ry matrix in the dentate neuroepithelium (Figure 6E).

Given the described consequences derived from Sox5 developmental loss in DG development, we wondered if Sox6 could also play a similar role at these stages. To explore this possibility, we generated a conditional Sox6^fl/fl^ line (Dumitriu et al., 2006) crossed to a Nestin-cre line (Figure 6H; Sox6^null^). Analysis of cell proliferation in the DG in P20 mice did not reveal any differences in the number of Ki67^+^ cells (318.10 ± 62.64 cells/section in Control vs. 233.3 ± 40.32 cells/section in Sox6^null^; Figures 6I,J) or BrdU^+^ cells between Control and Sox6^null^ mice (128.6 ± 29.09 cells/section and 134.5 ± 30.19 cells/section, respectively; Figures 6I,K).

These results indicate that Sox6, like Sox5, is expressed in NSCs and early progenitors during DG development, and that its expression decays with neural commitment. However, in contrast with Sox5, embryonic loss of Sox6 apparently does not alter the proliferation of neural progenitors in the DG.

### Heterozygous loss of Sox6 in Sox5-deficient mice leads to profound alterations in DG morphology and NSC and IPC proliferation

**FIGURE 7:**
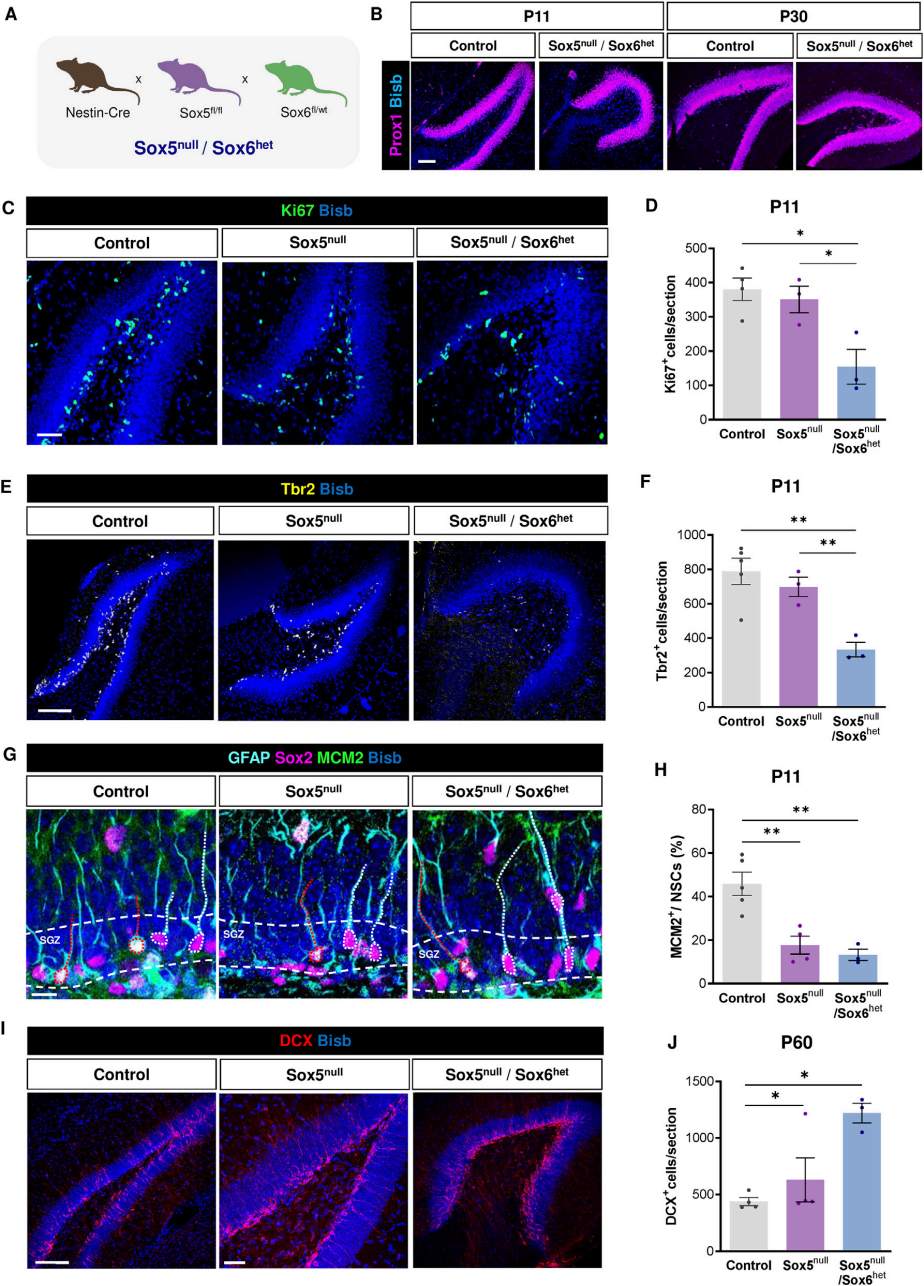
Heterozygous loss of Sox6 in Sox5-deficient mice leads to profound alterations in DG morphology and in NSC and IPC proliferation. **(A)** Generation of Sox5^null^/Sox6^het^ mice. **(B)** Confocal images showing DG morphology following Prox1 staining of Control and Sox5^null^/ Sox6^het^ mice at P11 and P30. **(C)** Confocal images showing Ki67^+^ proliferative cells in the DG of Control, Sox5^null^ and Sox5^null^/Sox6^het^ P11 mice. **(D)** Quantitation of Ki67^+^ proliferative cells in the DG per section. **(E)** Confocal images showing Tbr2^+^ IPCs in the DG of Control, Sox5^null^ and Sox5^null^/Sox6^het^ P11 mice. **(F)** Quantitation of Tbr2^+^ IPCs in the DG per section. **(G)** Confocal images showing MCM2^+^ proliferative NSCs (GFAP^+^ Sox2^+^) in the SGZ of Control, Sox5^null^ and Sox5^null^/Sox6^het^ P11 mice. **(H)** Quantitation of the % of MCM2^+^ proliferative NSCs (GFAP^+^ Sox2^+^ MCM2^+^ over total NSCs) in the SGZ. **(I)** Confocal images showing DCX^+^ immature GNs in the DG of Control, Sox5^null^ and Sox5^null^/Sox6^het^ P60 mice. **(J)** Quantitation of DCX^+^ cells in the DG per section. At least 3 animals were analyzed for each group. In all graphs, data are mean value ± SEM. *p < 0.05; **p < 0.01 by ordinary one-way ANOVA. Scale bars represent 100 µm (B, E, I Control and Sox5^null^/ Sox6^het^), 50 µm (C, I Sox5^null^) and 10 µm **(G)**.

To determine the possible contribution of Sox6 in the context of DG development in Sox5^null^ mice, we generated conditional mutant mice with homozygous loss of *Sox5 (*Sox5^fl/fl^) and heterozygous loss of *Sox6* (Sox6^fl/wt^) under the control of the Nestin promoter (Sox5^null^/Sox6^het^; Figure 7A).

Unlike Sox5 or Sox6 mutants, Sox5^null^/Sox6^het^ mice showed profound alterations in DG morphology and shape, both in postnatal and adult stages (Figure 7B). Analysis of cell proliferation using Ki67 marker as early as P11 revealed a marked decreased in Ki67^+^ cells in Sox5^null^/Sox6^het^ animals (154.2 ± 50.52 cells/section) compared to both Control and Sox5^null^ mice (379.9 ± 33.21 cells/section, *P* = 0.0121 and 350.5 ± 38.94 cells/section, *P* = 0.0318, respectively; Figures 7C,D). Note that in P11 Sox5^null^ mice differences in proliferation were not yet observed. Moreover, a more dramatic reduction in the number of Tbr2^+^ cells was found in Sox5^null^/Sox6^het^ mutants, with a decrease in IPC numbers of 42.5% and 47.7% compared to Control and Sox5^null^ mice, respectively (788.8 ± 75.57 in Control vs. 698.3 ± 57.19 cells/section in Sox5^null^ vs. 333.3 ± 41.74 cells/section in Sox5^null^/Sox6^het^; *P* = 0.0042 and *P* = 0.0252, respectively; Figures 7E,F). Finally, we assessed NSC proliferation, as this aspect is altered in postnatal Sox5^null^ mice (Medina-Menéndez et al., 2025). Proliferating NSCs at P11 were identified as GFAP^+^ Sox2^+^ cells with radial morphology and MCM2 labelling (cell cycle marker). We observed that Sox5^null^/Sox6^het^ mutants exhibited similar reduction in active NSCs as Sox5^null^ animals (45.86% ± 5.36% in Control vs. 17.69% ± 4.11% in Sox5^null^ vs. 13.19% ± 2.61% in Sox5^null^/Sox6^het^; *P* = 0.0044 and *P* = 0.0029, respectively; Figures 7G,H), suggesting a more prominent role for Sox5 in the context of NSC proliferation/activation. To analyse the possible consequences on neurogenesis, we analysed DCX^+^ immature neurons in adult mice of the different genotypes. In line with previous results, we observed a more accused phenotype in Sox5^null^/Sox6^het^ mice, which presented a 177% increase in DCX^+^ immature neurons compared to Control animals (438.6% ± 35.04% in Control vs. 631.0% ± 194.8% in Sox5^null^ vs. 1220% ± 86.41% in Sox5^null^/Sox6^het^; *P* = 0.0382 and *P* = 0.00293 respectively; Figures 7I,J).

Overall, these results indicate that combined loss of Sox5 (total) and Sox6 (partial) in mice aggravates the proliferation and differentiation defects and impaired DG development observed in Sox5^null^ mice, indicating a clear contribution of Sox6 to the control of neurogenesis during DG postnatal development.

## Discussion

DG development is under the tight control of signals that operate at different and often multiple steps of the neurogenic cascade, ensuring the correct establishment of its cell populations and morphology. Proliferating neural progenitors in the developing DG are mostly NSCs and IPCs. Although several studies have focused on the dynamics of neural progenitors as a whole or on NSCs as the cell population that acquires the quiescent state, it is less clear what are the mechanisms controlling cell cycle length and exit of the IPCs. Sox5 and Sox6 are TFs expressed in NSCs and IPCs of the DG and have been described as critical for the control of quiescence-activation dynamics in NSCs of the adult (both Sox5 and Sox6) and developmental DG (only Sox5; Li et al., 2022; Medina-Menéndez et al., 2025). Now, we describe that embryonic loss of Sox5 provokes subtle but distinctive changes in DG morphology in young adult mice, which are in part due to both an increase in IPC number and GN production and a reduction in cell death. Moreover, Sox5 loss shortens S-phase length in IPCs, probably contributing to the generation of aberrant immature neurons with altered location within the GCL and co-expression of DCX and NeuN proteins. In addition, we describe Sox6 contribution, together with Sox5, to the generation of the correct number of IPCs during early postnatal DG development. In summary, our data suggest that Sox5 and Sox6 act as stage-dependent regulators of neural progenitor cell cycle dynamics during DG development, exerting distinct effects at different levels of the neurogenic cascade.

The DG is a unique brain structure as it undergoes significant development after birth and continues to produce new neurons throughout life. The size and cellular density of the DG are controlled by a precise balance between neural progenitor cell proliferation, migration, and survival through a specific interplay between TFs and signaling pathways. For instance, Wnt signaling pathway, particularly through ligands such as Wnt3a and TFs as Lef1, drives the massive expansion of neural progenitors during the first weeks of life (Lie et al., 2005; Galceran et al., 2000). Similarly, loss of TFs such as Sox2 causes a severe reduction in DG size due to NSC loss and neural progenitor cell death (Favaro et al., 2009). By contrast, TFs such as Tbr2 (Eomes) and COUP-TFI (NR2F1) regulate the divisions of IPCs, ensuring a sufficient cellular base to reach adult structural volume (Hodge et al., 2012; Parisot et al., 2017). In the case of Sox5 loss, we have found subtle but significant alterations in the DG morphology and size upon conditional Sox5 loss. The main structural changes are observed at P30, when there is an increase in GCL thickness and a shortening of the infrapyramidal blade length that is accompanied by bigger angle between DG blades. These morphological alterations are likely due to the observed overproduction of immature neurons and GNs in Sox5^null^ mice by P30, combined by a decrease in the number of apoptotic cells. In the DG, cells undergo two waves of apoptosis. The first one affects late IPCs in their transition to immature neurons and the second is associated to the maturation process, when cells begin to express NeuN and Prox1 (Sierra et al., 2010). Therefore, it is possible that the differentiation defects observed in Sox5^null^ mice could be in part linked to the reduction in cell death reported in these animals.

On the other side, there is an abnormal distribution of immature neurons in the upper part of granular layer, closer to the molecular cell layer, that could contribute to the abnormal shape of the DG. Similar defects have been reported for COUP-TF1, a TF that shares functions with Sox5 during neural development (Bertacchi et al., 2019). Loss of COUP-TF1 provokes, like in the case of Sox5 loss, a general reduction in progenitor proliferation. However, the defects in COUP-TF1 mutants appear during embryonic stages, whereas in Sox5^null^ mice, general proliferation defects are only apparent from P14 onward. Moreover, COUP-TF1 loss provokes an increase in apoptosis and a decrease in IPC proliferation, whereas Sox5 loss enhances survival and does not affect the proliferating fraction of IPCs. Furthermore, unlike what has been observed following Sox5 loss, all neural progenitors exhibit a premature cell cycle exit in COUP-TF1 mutants. Nevertheless, in both cases early changes in cell cycle dynamics contributes to premature and aberrant differentiation, reflected in the increase of neurons co-expressing Tbr2/Prox1 (Parisot et al., 2017) or DCX/NeuN (400-fold increase in our data). Sox5 expression is also found in the CH and CA regions during embryonic development. Despite this additional expression pattern, it is very unlikely that CA cells could contribute to neural progenitor cell cycle regulation in the DG, as they originate from different neuroepithelial regions and differentiate in distinct developmental windows. However, the role of Sox5 expressing astrocytes in DG neurogenesis regulation remains to be explored. Thus, in contrast with other TFs that modulate general growth during DG development, Sox5 is required for the specific control of NSC quiescence (Medina-Menéndez et al., 2025) and to control S-phase length in IPCs (data in this work).

The role of Sox5 in the control of cell cycle dynamics varies depending on the cellular context. Thus, in neural progenitors of the chick spinal cord and in the adult subventricular zone, Sox5 promotes cell cycle exit (Martinez-Morales et al., 2010; Kurtsdotter et al., 2017). The results described here for the developing DG do not point to a role of Sox5 in promoting cell cycle exit. These seemingly opposite functions probably depend on the specific transcriptional programs and TFs interacting with Sox5 in each case. Moreover, as Tbr2^+^ IPCs are mostly mitotic progenitors, our analysis of the cell cycle exit index (Ki67^-^/BrdU^+^Tbr2^+^) could be also interpreted as a shortening of cell cycle duration. In fact, when using the dual thymidine analog labelling, we unraveled a significant shortening of the S-phase in Sox5^null^ IPCs at P14. As cell cycle length has been described as an important aspect in cell fate decisions, and specially G1 and S-phase duration may have a crucial role in the precursor maintenance versus differentiation decision (Hardwick and Philpott, 2014), these alterations in Sox5^null^ IPCs cell cycle dynamics could be responsible for the GN maturation defects observed. Another case of context-dependent Sox5 function is the fact that Sox5 directly controls the *Ascl1* transcription to promote adult NSCs activation (Li et al., 2022). However, Ascl1 is not required for NSC proliferation during postnatal stages or in general for DG development (Andersen et al., 2014).

An important matter that arises with these results is the contribution of Sox5 function in NSCs activation versus the control in IPC cell cycle dynamics in the context of DG neurogenesis. As we had previously described, Sox5^null^ mice show an impaired acquisition of NSC quiescence depth during postnatal DG development. In P14 Sox5 mutants, NSCs accumulate in a superficial primed quiescent state at the expense of reducing the fraction of proliferating NSCs. At P30, Sox5^null^ mice present an over activation of the NSC pool, a direct consequence of the imbalance in NSC quiescence depth, and, finally, a premature depletion of the NSCs in the adult DG is observed at P90 (Medina-Menéndez et al., 2025). The changes in NSC activation observed in Sox5-deficient mice mirror both the initial decrease in Tbr2^+^ IPCs and DCX^+^ immature neurons at P14 that we describe in this work, which could be partly due to the reduction in NSC activation, and the increase in these two populations at P30, when we see the opposite activation pattern in NSCs. Therefore, these changes in principle could be attributed to the differences in NSC activation. The defects in IPC cell cycle progression, however, remain the same between P14 and P30 stages, which suggests that there is an extra layer of regulation in the final number of new neurons generated in the developing DG that is exerted through the control of IPC cell cycle by Sox5. In spite of this, Tbr2^+^ and DCX^+^ cells final numbers in Sox5^null^ mice follow the pattern of NSC proliferation despite the changes in IPC cell cycle progression, which highlights the importance of NSCs dynamics for neurogenesis. It is worthwhile mentioning that NSCs are considered the main driver of neurogenesis compared to more differentiated neural populations, that exert a weaker influence in adult neurogenesis regulation (Danciu et al., 2025). Nevertheless, IPC cell cycle duration could be very relevant for the correct differentiation program of the generated neurons and the shortening of S-phase could be behind the defects in new neurons location and maturation observed in Sox5^null^ mice. Furthermore, those immature neurons at the misplaced position could establish wrong connections with inappropriate nearby targets in the neural network. There are other cell types in which Sox5 has been reported to prevent early differentiation. For instance, Sox5 deletion in oligodendrocyte precursor cells induces the premature appearance of differentiated oligodendrocyte (Stolt et al., 2006), while its overexpression delays fully chondrogenic differentiation (Sun et al., 2015).

Finally, we have characterized Sox6 expression during DG development, which is restricted to neural progenitors, predominantly NSCs, and is absent in differentiated cells. Thus, both in developmental and adult DG, Sox5 and Sox6 shared a very similar expression pattern (Li et al., 2022). This expression pattern has also been described in the adult subventricular zone (SVZ) neurogenic niche (Kurtsdotter et al., 2017). In spite of these similarities, developmental loss of Sox6 does not recapitulate the DG alterations here described for Sox5^null^ mice, whereas homozygous *Sox5* loss combined with one *Sox6* allele loss leads to important defects in DG morphology and a more severe phenotype than the one observed with Sox5 loss alone. In this case, Sox5^null^Sox6^het^ mice show a sharp decline in Ki67^+^ and Tbr2^+^ progenitors but not in NSC proliferation. Together, these results suggest that Sox6 may be partially dispensable for the control of proliferation in developing NSCs, whereas it has a more prominent role in IPC generation and in neurogenesis. Therefore, our results point out to partially redundant and synergistic roles of Sox5 and Sox6 during DG development, particularly in IPCs. This is a common feature found amongst Sox genes (Kamachi and Kondoh, 2013) and it has been described for Sox5 and Sox6 in chondrocytes (Smits et al., 2001; Ikeda et al., 2004), oligodendrocytes (Stolt et al., 2006) and in adult NSCs in the DG (Li et al., 2022) and in the SVZ upon oncogenic stress (Kurtsdotter et al., 2017).

In humans, mutations in *SOX5* and *SOX6* are the cause of recently described rare neurodevelopmental syndromes (Lamb et al., 2012; Tolchin et al., 2020; Tenorio-Castano et al., 2023). Therefore, uncovering SoxD function in neural development would be crucial to fully understand the pathophysiology of these rare diseases and, consequently, advance in the discovery of therapeutical interventions.

## Data Availability

The raw data supporting the conclusions of this article will be made available by the authors, without undue reservation.

## Supplementary material

SUPPLEMENTARY FIGURE S1 Loss of Sox5 expression causes subtle changes in DG morphology in P30 mice. **(A)** Nissl staining images showing brain morphology of Control and Sox5^null^ mice at the indicated stages. Scale bars represent 250 µm in P0 and 300 µm in P5-P30.

## References

[B1] Altman J. Bayer S. A. (1990). Migration and distribution of two populations of hippocampal granule cell PrecursorsDurrng the perinatal and postnatal periods. J. Comp. Neurology 301 (3), 365–381. 10.1002/cne.903010304 2262596

[B2] Altman J. Das G. D. (1965). Autoradiographic and histological evidence of postnatal hippocampal neurogenesis in rats. J. Comp. Neurology 124 (3), 319–335. 10.1002/cne.901240303 5861717

[B3] Andersen J. Urbán N. Achimastou A. Ito A. Simic M. Ullom K. (2014). A transcriptional mechanism integrating inputs from extracellular signals to activate hippocampal stem cells. Neuron 83 (5), 1085–1097. 10.1016/j.neuron.2014.08.004 25189209 PMC4157576

[B4] Bayer S. A. Altman J. (1974). Hippocampal development in the rat: cytogenesis and morphogenesis examined with autoradiography and low-level X-irradiation. J. Comp. Neurology 158 (1), 55–79. 10.1002/cne.901580105 4430737

[B5] Beccari S. Diaz‐Aparicio I. Sierra A. (2018). Quantifying microglial phagocytosis of apoptotic cells in the brain in health and disease. Curr. Protoc. Immunol. 122 (1), e49. 10.1002/cpim.49 29927067

[B6] Berg D. A. Su Y. Jimenez-Cyrus D. Patel A. Huang N. Morizet D. (2019). A common embryonic origin of stem cells drives developmental and adult neurogenesis. Cell 177 (3), 654–668.e15. 10.1016/j.cell.2019.02.010 30929900 PMC6496946

[B7] Bertacchi M. Parisot J. Studer M. (2019). The pleiotropic transcriptional regulator COUP-TFI plays multiple roles in neural development and disease. Brain Res. 1705, 75–94. 10.1016/j.brainres.2018.04.024 29709504

[B8] Bond A. M. Ming G. Song H. (2021). “Ontogeny of adult neural stem cells in the Mammalian brain,” in Current Topics in Developmental Biology. Editor Bashaw G. J. (Academic Press Inc), 142, 67–98. 10.1016/bs.ctdb.2020.11.002 PMC8363052 33706926

[B9] Brandt M. D. Hübner M. Storch A. (2012). Brief report: adult hippocampal precursor cells shorten S-Phase and total cell cycle length during neuronal differentiation. Stem Cells 30 (12), 2843–2847. 10.1002/stem.1244 22987479

[B10] Calatayud-Baselga I. Casares-Crespo L. Franch-Ibáñez C. Guijarro-Nuez J. Sanz P. Mira H. (2023). Autophagy drives the conversion of developmental neural stem cells to the adult quiescent state. Nat. Commun. 14 (1), 7541. 10.1038/s41467-023-43222-1 38001081 PMC10673888

[B11] Caronia G. Wilcoxon J. Feldman P. Grove E. A. (2010). Bone morphogenetic protein signaling in the developing telencephalon controls formation of the hippocampal dentate gyrus and modifies fear-related behavior. J. Neurosci. 30 (18), 6291–6301. 10.1523/JNEUROSCI.0550-10.2010 20445055 PMC2905858

[B12] Choe Y. Kozlova A. Graf D. Pleasure S. J. (2013). Bone morphogenic protein signaling is a major determinant of dentate development. J. Neurosci. 33 (16), 6766–6775. 10.1523/JNEUROSCI.0128-13.2013 23595735 PMC3684166

[B13] Costa M. R. Ortega F. Brill M. S. Beckervordersandforth R. Petrone C. Schroeder T. (2011). Continuous live imaging of adult neural stem cell division and lineage progression *in vitro* . Development 138 (6), 1057–1068. 10.1242/dev.061663 21343361

[B14] Danciu D. P. Klawe F. Z. Kazarnikov A. Femmer L. Kostina E. Martin-Villalba A. (2025). Unraveling regulatory feedback mechanisms in adult neurogenesis through mathematical modelling. NPJ Syst. Biol. Appl. 11 (1), 82. 10.1038/s41540-025-00563-5 40715178 PMC12297322

[B15] Dumitriu B. Dy P. Smits P. Lefebvre V. (2006). Generation of mice harboring a Sox6 conditional null allele. Genesis 44 (5), 219–224. 10.1002/dvg.20210 16652367

[B16] Dy P. Han Y. Lefebvre V. (2008). Generation of mice harboring a Sox5 conditional null allele. Genesis 46 (6), 294–299. 10.1002/dvg.20392 18543318 PMC11783625

[B17] Favaro R. Valotta M. Ferri A. L. M. Latorre E. Mariani J. Giachino C. (2009). Hippocampal development and neural stem cell maintenance require Sox2-dependent regulation of shh. Nat. Neurosci. 12 (10), 1248–1256. 10.1038/nn.2397 19734891

[B18] Flore G. di Ruberto G. Parisot J. Sannino S. Russo F. Illingworth E. A. (2017). Gradient COUP-TFI expression is required for functional organization of the hippocampal septo-temporal longitudinal axis. Cereb. Cortex 27 (2), 1629–1643. 10.1093/cercor/bhv336 26813976

[B19] Franz T. (1994). Extra-toes (xt) homozygous mutant mice demonstrate a role for the Gli-3 gene in the development of the forebrain. Acta Anat. 150 (1), 38–44. 10.1159/000147600 7976186

[B20] Galceran J. Miyashita-Lin E. M. Devaney E. Rubenstein J. L. R. Grosschedl R. (2000). Hippocampus development and generation of dentate gyrus granule cells is regulated by LEF1. Development 127 (3), 469–482. 10.1242/dev.127.3.469 10631168

[B21] Galeeva A. Treuter E. Tomarev S. Pelto-Huikko M. (2007). A prospero-related homeobox gene Prox-1 is expressed during postnatal brain development as well as in the adult rodent brain. Neuroscience 146 (2), 604–616. 10.1016/j.neuroscience.2007.02.002 17368742

[B22] Galichet C. Guillemot F. Parras C. M. (2008). Neurogenin 2 has an essential role in development of the dentate gyrus. Development 135 (11), 2031–2041. 10.1242/dev.015115 18448566

[B23] Grove E. A. Tole S. Limon J. Yip L. Ragsdale C. W. (1998). The hem of the embryonic cerebral cortex is defined by the expression of multiple wnt genes and is compromised in Gli3-deficient mice. Development 125 (12), 2315–2325. 10.1242/dev.125.12.2315 9584130

[B24] Hardwick L. J. A. Philpott A. (2014). Nervous decision-making: to divide or differentiate. Trends Genet. 30 (6), 254–261. 10.1016/j.tig.2014.04.001 24791612 PMC4046230

[B25] Hardwick L. J. A. Ali F. R. Azzarelli R. Philpott A. (2015). Cell cycle regulation of proliferation versus differentiation in the central nervous system. Cell Tissue Res. 359 (1), 187–200. 10.1007/s00441-014-1895-8 24859217 PMC4284380

[B26] Hasenpusch-Theil K. Magnani D. Amaniti E. M. Han L. Armstrong D. Theil T. (2012). Transcriptional analysis of Gli3 mutants identifies Wnt target genes in the developing hippocampus. Cereb. Cortex 22 (12), 2878–2893. 10.1093/cercor/bhr365 22235033 PMC3491769

[B27] Hatami M. Conrad S. Naghsh P. Alvarez-Bolado G. Skutella T. (2018). Cell-biological requirements for the generation of dentate gyrus granule neurons. Front. Cell. Neurosci. 12, 402. 10.3389/fncel.2018.00402 30483057 PMC6240695

[B28] Heng Y. H. E. McLeay R. C. Harvey T. J. Smith A. G. Barry G. Cato K. (2014). NFIX regulates neural progenitor cell differentiation during hippocampal morphogenesis. Cereb. Cortex 24 (1), 261–279. 10.1093/cercor/bhs307 23042739 PMC3862270

[B29] Hodge R. D. Nelson B. R. Kahoud R. J. Yang R. Mussar K. E. Reiner S. L. (2012). Tbr2 is essential for hippocampal lineage progression from neural stem cells to intermediate progenitors and neurons. J. Neurosci. 32 (18), 6275–6287. 10.1523/JNEUROSCI.0532-12.2012 22553033 PMC3366485

[B30] Hodge R. D. Garcia A. J. Elsen G. E. Nelson B. R. Mussar K. E. Reiner S. L. (2013). Tbr2 expression in Cajal-Retzius cells and intermediate neuronal progenitors is required for morphogenesis of the dentate gyrus. J. Neurosci. 33 (9), 4165–4180. 10.1523/JNEUROSCI.4185-12.2013 23447624 PMC3623668

[B31] Ikeda T. Kamekura S. Mabuchi A. Kou I. Seki S. Takato T. (2004). The combination of SOX5, SOX6, and SOX9 (the SOX trio) provides signals sufficient for induction of permanent cartilage. Arthritis Rheumatism 50 (11), 3561–3573. 10.1002/art.20611 15529345

[B32] Iwano T. Masuda A. Kiyonari H. Enomoto H. Matsuzaki F. (2012). Prox1 postmitotically defines dentate gyrus cells by specifying granule cell identity over CA3 pyramidal cell fate in the hippocampus. Development 139 (16), 3051–3062. 10.1242/dev.080002 22791897

[B33] Kamachi Y. Kondoh H. (2013). Sox proteins: regulators of cell fate specification and differentiation. Dev. Camb. 140 (20), 4129–4144. 10.1242/dev.091793 24086078

[B34] Kee N. Sivalingam S. Boonstra R. Wojtowicz J. M. (2002). The utility of Ki-67 and BrdU as proliferative markers of adult neurogenesis. J. Neurosci. Methods 115 (1), 97–105. 10.1016/s0165-0270(02)00007-9 11897369

[B35] Kurtsdotter I. Topcic D. Karlén A. Singla B. Hagey D. W. Bergsland M. (2017). SOX5/6/21 prevent oncogene-driven transformation of brain stem cells. Cancer Res. 77 (18), 4985–4997. 10.1158/0008-5472.CAN-17-0704 28687615 PMC11783646

[B36] Lamb A. N. Rosenfeld J. A. Neill N. J. Talkowski M. E. Blumenthal I. Girirajan S. (2012). Haploinsufficiency of SOX5 at 12p12.1 is associated with developmental delays with prominent language delay, behavior problems, and mild dysmorphic features. Hum. Mutat. 33 (4), 728–740. 10.1002/humu.22037 22290657 PMC3618980

[B37] Lavado A. Lagutin O. v. Chow L. M. L. Baker S. J. Oliver G. (2010). Prox1 is required for granule cell maturation and intermediate progenitor maintenance during brain neurogenesis. PLoS Biol. 8 (8), e1000460. 10.1371/journal.pbio.1000460 20808958 PMC2923090

[B38] Lee S. M. K. Tole S. Grove E. McMahon A. P. (2000). A local Wnt-3a signal is required for development of the mammalian hippocampus. Development 127 (3), 457–467. 10.1242/dev.127.3.457 10631167

[B39] Lefebvre V. (2010). The SoxD transcription factors - Sox5, Sox6, and Sox13 - are key cell fate modulators. Int. J. Biochem. Cell Biol. 42 (3), 429–432. 10.1016/j.biocel.2009.07.016 19647094 PMC2826538

[B40] Lefebvre V. Dumitriu B. Penzo-Méndez A. Han Y. Pallavi B. (2007). Control of cell fate and differentiation by Sry-related high-mobility-group Box (Sox) transcription factors. Int. J. Biochem. Cell Biol. 39 (12), 2195–2214. 10.1016/j.biocel.2007.05.019 17625949 PMC2080623

[B41] Li L. Medina-Menéndez C. García-Corzo L. Córdoba-Beldad C. M. Quiroga A. C. Calleja Barca E. (2022). SoxD genes are required for adult neural stem cell activation. Cell Rep. 38 (5), 110313. 10.1016/j.celrep.2022.110313 35108528 PMC11783645

[B42] Lie D. C. Colamarino S. A. Song H. J. Désiré L. Mira H. Consiglio A. (2005). Wnt signalling regulates adult hippocampal neurogenesis. Nature 437 (7063), 1370–1375. 10.1038/nature04108 16251967

[B43] Liu M. Pleasure S. J. Collins A. E. Noebels J. L. Naya F. J. Tsai M.-J. (2000). Loss of BETA2/NeuroD leads to malformation of the dentate gyrus and epilepsy. PNAS 97 (2), 865–870. 10.1073/pnas.97.2.865 10639171 PMC15422

[B44] Llorens-Martín M. Tejeda G. S. Trejo J. L. (2010). Differential regulation of the variations induced by environmental richness in adult neurogenesis as a function of time: a dual birthdating analysis. PLoS ONE 5 (8), e12188. 10.1371/journal.pone.0012188 20808440 PMC2922333

[B45] Martinez-Morales P. L. Quiroga A. C. Barbas J. A. Morales A. V. (2010). SOX5 controls cell cycle progression in neural progenitors by interfering with the WNT-Β-catenin pathway. EMBO Rep. 11 (6), 466–472. 10.1038/embor.2010.61 20448664 PMC2892326

[B46] Medina-Menéndez C. Tirado-Melendro P. Li L. Rodríguez-Martín P. Melgarejo-De la Peña E. Díaz-García M. (2025). Sox5 controls the establishment of quiescence in neural stem cells during postnatal development. PLoS Biol. 23 (7), e3002654. 10.1371/journal.pbio.3002654 40720543 PMC12321142

[B47] Miller I. Min M. Yang C. Tian C. Gookin S. Carter D. (2018). Ki67 is a graded rather than a binary marker of proliferation versus quiescence. Cell Rep. 24 (5), 1105–1112.e5. 10.1016/j.celrep.2018.06.110 30067968 PMC6108547

[B48] Miyata T. Maeda T. Lee J. E. (1999). NeuroD is required for differentiation of the granule cells in the cerebellum and hippocampus. Genes Dev. 13 (13), 1647–1652. 10.1101/gad.13.13.1647 10398678 PMC316850

[B49] Morales A. v. Mira H. (2019). Adult neural stem cells: born to last. Front. Cell Dev. Biol. 7 (JUN), 96. 10.3389/fcell.2019.00096 31214589 PMC6557982

[B50] Noguchi H. Garcia Castillo J. Nakashima K. Pleasure S. J. (2019). Suppressor of fused controls perinatal expansion and quiescence of future dentate adult neural stem cells. eLife 8 (8), e42918. 10.7554/eLife.42918.001 30973324 PMC6459675

[B51] Nowakowski R. S. Caviness V. S. Takahashi T. Hayes N. L. (2002). Population dynamics during cell proliferation and neuronogenesis in the developing murine neocortex. In: Cortical Development. Results and Problems in Cell Differentiation, Hohmann C. 39. 1, 25. Berlin, Heidelberg: Springer. 10.1007/978-3-540-46006-0_1 12353465

[B52] Odin J. A. Huebert R. C. Casciola-Rosen L. LaRusso N. F. Rosen A. (2001). Bcl-2-dependent oxidation of pyruvate dehydrogenase-E2, a primary biliary cirrhosis autoantigen, during apoptosis. J. Clin. Invest. 108 (2), 223–232. 10.1172/JCI10716 11457875 PMC203018

[B53] Oishi S. Premarathne S. Harvey T. J. Iyer S. Dixon C. Alexander S. (2016). Usp9x-deficiency disrupts the morphological development of the postnatal hippocampal dentate gyrus. Sci. Rep. 6, 25783. 10.1038/srep25783 27181636 PMC4867638

[B54] Parisot J. Flore G. Bertacchi M. Studer M. (2017). COUP-TFI mitotically regulates production and migration of dentate granule cells and modulates hippocampal Cxcr4 expression. Development 144 (11), 2045–2058. 10.1242/dev.139949 28506990

[B55] Pastor-Alonso O. Syeda Zahra A. Kaske B. García-Moreno F. Tetzlaff F. Bockelmann E. (2024). Generation of adult hippocampal neural stem cells occurs in the early postnatal dentate gyrus and depends on cyclin D2. EMBO J. 43 (3), 317–338. 10.1038/s44318-023-00011-2 38177500 PMC10897295

[B56] Ponti G. Obernier K. Guinto C. Jose L. Bonfanti L. Alvarez-Buylla A. (2013). Cell cycle and lineage progression of neural progenitors in the ventricular-subventricular zones of adult mice. PNAS 110 (11), E1045–E1054. 10.1073/pnas.1219563110 23431204 PMC3600494

[B57] Roybon L. Hjalt T. Stott S. Guillemot F. Li J. Y. Brundin P. (2009). Neurogenin2 directs granule neuroblast production and amplification while neuroD1 specifies neuronal fate during hippocampal neurogenesis. PLoS ONE 4 (3), e4779. 10.1371/journal.pone.0004779 19274100 PMC2652712

[B58] Sierra A. Encinas J. M. Deudero J. J. P. Chancey J. H. Enikolopov G. Overstreet-Wadiche L. S. (2010). Microglia shape adult hippocampal neurogenesis through apoptosis-coupled phagocytosis. Cell Stem Cell 7 (4), 483–495. 10.1016/j.stem.2010.08.014 20887954 PMC4008496

[B59] Simon R. Brylka H. Schwegler H. Venkataramanappa S. Andratschke J. Wiegreffe C. (2012). A dual function of Bcl11b/Ctip2 in hippocampal neurogenesis. EMBO J. 31 (13), 2922–2936. 10.1038/emboj.2012.142 22588081 PMC3395096

[B60] Smits P. Li P. Mandel J. Zhang Z. Deng M. J. Behringer R. R. (2001). The transcription factors L-Sox5 and Sox6 are essential for cartilage formation. Dev. Cell 1 (2), 277–290. 10.1016/s1534-5807(01)00003-x 11702786

[B61] Snyder J. S. (2019). Recalibrating the relevance of adult neurogenesis. Trends Neurosci. 42 (3), 164–178. 10.1016/j.tins.2018.12.001 30686490

[B62] Stolt C. C. Schlierf A. Lommes P. Hillgärtner S. Werner T. Kosian T. (2006). SoxD proteins influence multiple stages of oligodendrocyte development and modulate SoxE protein function. Dev. Cell 11 (5), 697–709. 10.1016/j.devcel.2006.08.011 17084361

[B63] Sun D. Deepak V. Mu P. Jiang H. Shi X. Liu Z. (2015). Constitutive L-Sox5 overexpression delays differentiation of ATDC5 cells into chondrocytes and correlates with reduced expression of differentiation markers. Mol. Cell. Biochem. 401 (1–2), 21–26. 10.1007/s11010-014-2288-8 25445168

[B64] Tenorio-Castano J. Gómez Á. S. A. Coronado M. Rodríguez-Martín P. Parra A. Pascual P. (2023). Lamb–Shaffer syndrome: 20 Spanish patients and literature review expands the view of neurodevelopmental disorders caused by SOX5 haploinsufficiency. Clin. Genet. 104 (6), 637–647. 10.1111/cge.14423 37702321

[B65] Tolchin D. Yeager J. P. Prasad P. Dorrani N. Russi A. S. Martinez-Agosto J. A. (2020). *De novo* SOX6 Variants Cause a Neurodevelopmental Syndrome Associated with ADHD, Craniosynostosis, and Osteochondromas. Am. J. Hum. Genet. 106 (6), 830–845. 10.1016/j.ajhg.2020.04.015 32442410 PMC7273536

[B66] Unal-Cevik I. Kilinç M. Gürsoy-Ozdemir Y. Gurer G. Dalkara T. (2004). Loss of NeuN immunoreactivity after cerebral ischemia does not indicate neuronal cell loss: a cautionary note. Brain Res. 1015 (1-2), 169–174. 10.1016/j.brainres.2004.04.032 15223381

[B67] Urbán N. Guillemot F. (2014). Neurogenesis in the embryonic and adult brain: same regulators, different roles. Front. Cell. Neurosci. 8 (NOV), 396. 10.3389/fncel.2014.00396 25505873 PMC4245909

[B68] Vithayathil J. Pucilowska J. Goodnough L. H. Atit R. P. Landreth G. E. (2015). Dentate gyrus development requires ERK activity to maintain progenitor population and MAPK pathway feedback regulation. J. Neurosci. 35 (17), 6836–6848. 10.1523/JNEUROSCI.4196-14.2015 25926459 PMC4412899

[B69] Zhou C. J. Zhao C. Pleasure S. J. (2004). Wnt signaling mutants have decreased dentate granule cell production and radial glial scaffolding abnormalities. J. Neurosci. 24 (1), 121–126. 10.1523/JNEUROSCI.4071-03.2004 14715945 PMC6729560

